# Kinetics of Rhodopsin Deactivation and Its Role in Regulating Recovery and Reproducibility of Rod Photoresponse

**DOI:** 10.1371/journal.pcbi.1001031

**Published:** 2010-12-16

**Authors:** Giovanni Caruso, Paolo Bisegna, Leonardo Lenoci, Daniele Andreucci, Vsevolod V. Gurevich, Heidi E. Hamm, Emmanuele DiBenedetto

**Affiliations:** 1 Construction Technologies Institute, National Research Council, Rome, Italy; 2 Department of Civil Engineering, University of Rome Tor Vergata, Rome, Italy; 3 Department of Pharmacology, Vanderbilt University Medical Center, Nashville, Tennessee, United States of America; 4 Department of Mathematical Methods and Models, University of Rome La Sapienza, Rome, Italy; 5 Department of Mathematics, Vanderbilt University, Nashville, Tennessee, United States of America; UT Southwestern Medical Center, United States of America

## Abstract

The single photon response (SPR) in vertebrate phototransduction is regulated by the dynamics of R^*^ during its lifetime, including the random number of phosphorylations, the catalytic activity and the random sojourn time at each phosphorylation level. Because of this randomness the electrical responses are expected to be inherently variable. However the SPR is highly reproducible. The mechanisms that confer to the SPR such a low variability are not completely understood. The kinetics of rhodopsin deactivation is investigated by a Continuous Time Markov Chain (CTMC) based on the biochemistry of rhodopsin activation and deactivation, interfaced with a spatio-temporal model of phototransduction. The model parameters are extracted from the photoresponse data of both wild type and mutant mice, having variable numbers of phosphorylation sites and, with the same set of parameters, the model reproduces both WT and mutant responses. The sources of variability are dissected into its components, by asking whether a random number of turnoff steps, a random sojourn time between steps, or both, give rise to the known variability. The model shows that only the randomness of the sojourn times in each of the phosphorylated states contributes to the Coefficient of Variation (CV) of the response, whereas the randomness of the number of R^*^ turnoff steps has a negligible effect. These results counter the view that the larger the number of decay steps of R^*^, the more stable the photoresponse is. Our results indicate that R^*^ shutoff is responsible for the variability of the photoresponse, while the diffusion of the second messengers acts as a variability suppressor.

## Author Summary

Reception and transmission of biological stimuli such as vision, olfaction, taste, and hormone and neurotransmitter signal transduction, contain inherently variable components. Yet, biological functions are stable and reliable. For each signaling process, it is of interest to investigate the causes of variability and the mechanisms by which variability is mitigated to yield responses that reliably reflect the strength of the stimulus. We have investigated the variability of the single photon response in rod photoreceptors. A photon of light is captured by a receptor rhodopsin, and it goes through a series of biochemical states ending with a random shutoff. We have created a mathematical model of such a process, based on the recent biochemical findings on activation/deactivation, capable of reproducing the peculiar experimental features of visual trasduction both in wild type and genetically modified mice. We have found that the randomness of the time that rhodopsin sojourns in each of these biochemical states is the dominant cause of variability, whereas diffusion of molecules carrying the signal within the cell acts as variability mitigators.

## Introduction

In retinal rod photoreceptors, rhodopsin activated by photons of light, denoted by 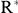, initiates a signal transduction cascade to produce a suppression of electrical current flowing into rod outer segment (ROS). Following isomerization, a molecule 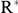 undergoes a random number of phosphorylations by rhodopsin kinase (RK) and finally is inactivated by arrestin (Arr) binding. Activated rhodopsin 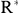, moving along its random path, during its random lifetime from isomerization to Arr binding, keeps activating its cognate G-protein (G) transducin, while its catalytic activity declines with increasing level of phosphorylation. The active G-protein (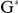) associates with the effector protein phosphodiesterase (E) forming an active 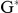-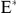 complex, which by hydrolyzing cGMP reduces its concentration, thereby generating a current response on the outer shell of the ROS. The dynamics of 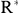 during its lifetime, including the random number of phosphorylations, the catalytic activity and the random sojourn time at each phosphorylation level, regulates the production of 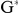 and therefore the current response. Because of the randomness in the components of the activation/deactivation cascade, the electrical responses are expected to be inherently variable. However, the single photon response (SPR) exhibits a low variability in the sense that the amplitude and shape of the electrical responses, corresponding to a set of activation-deactivation events, are similar. It is reported that the Coefficient of Variation (CV = standard deviation/mean) of the SPR area for mouse is about 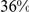 [1]. However, the mechanisms that confer high reproducibility of the SPR are not completely understood.

Several studies [1]–[7] attribute the high reproducibility of the SPR mainly to the mechanisms regulating rhodopsin deactivation. Although the models proposed in these studies account for the low variability of the response, they impose, in one way or another, certain restrictions on the biochemistry of rhodopsin deactivation. For example, if rhodopsin's integrated activity occurs in 
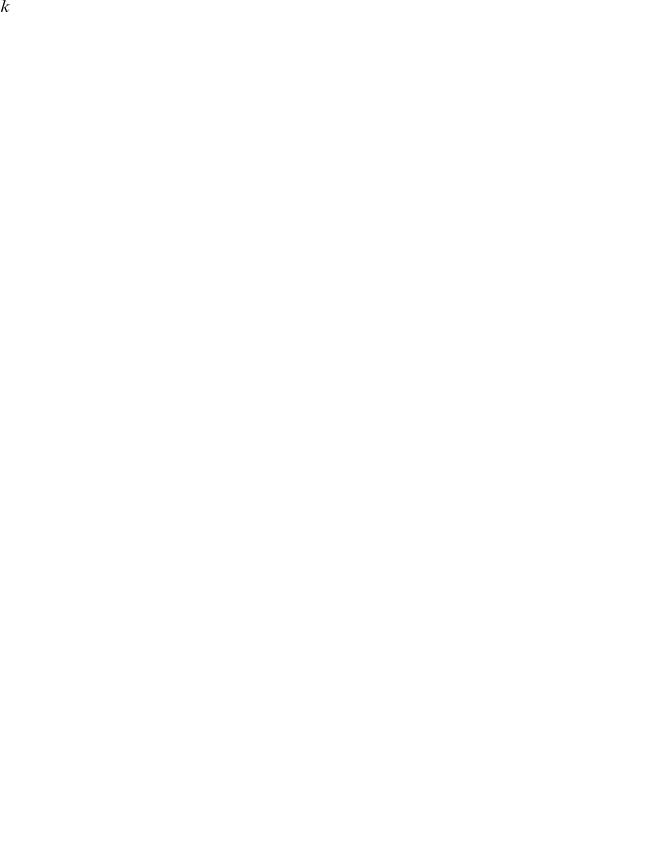
 independent steps, it is assumed that each step controls an equal fraction of rhodopsin's integrated catalytic activity [1], [2]. It is then natural to ask what is the statistical mean 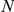 of the number 
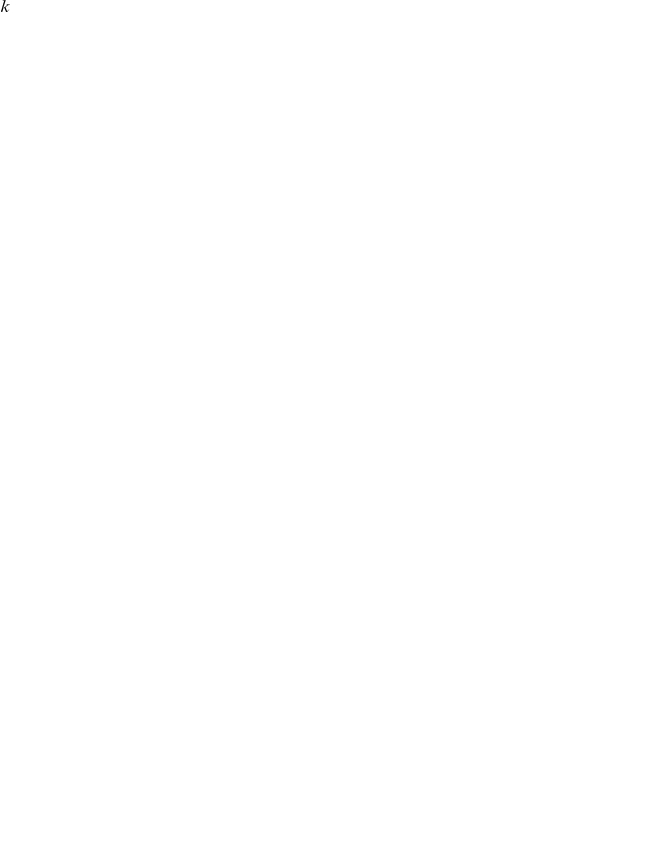
, as a way of testing both the models and the supporting biochemical assumptions. Mechanistically, one might ask which of the components of the deactivation cascade contribute more importantly to the variability.

A major difficulty with these issues is to experimentally separate the various components that contribute to the variability. To our knowledge, the activation/deactivation module of the cascade is not, to date, experimentally separable from the transduction module. We have shown in [8] that diffusion of the second messengers in the cytoplasm acts as a variability suppressor. The separation between the activation cascade on the disks and the diffusion of the second messengers cGMP and 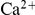 in the cytoplasm is realized by a mathematical model [8]–[11]. Likewise several fine properties of the biochemical and biophysical mechanisms regulating the recovery and reproducibility of SPR are not, to our knowledge, experimentally separable. Here we attempted to tease apart the various components of the 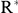 shutoff mechanism and analyze to what extent each of them contributes to the variability of the SPR. Unlike the transduction part of the cascade, where the intricacy is of geometrical nature [9]–[11], the main difficulty here is stochastic. Rhodopsin inactivation can occur by several mechanisms, including Arr binding and thermal decay to opsin. We only model the former, as the latter occurs on a much longer time scale [12], [13]. Shutoff of 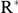 by Arr binding can follow, in principle, an infinite number of paths, depending on the random number of phosphorylated states, and the random sojourn times in those states.

The biochemistry that regulates rhodopsin deactivation is put into a stochastic framework, which reproduces the SPR both in WT and in mutant mice, and is capable of analyzing the randomness of each phosphorylation state of 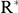. This is interfaced with the spatio-temporal model in [8], [9], [11], capable of tracking the diffusion of the second messengers in the cytoplasm and of detecting the effects of geometrical changes of the ROS on the photoresponse.

We find that the randomness of the sojourn times of 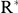 in each of its phosphorylation states acts as the dominant factor contributing to the CV of the response. At the same time the number of available phosphorylation sites or the random number of 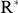 phosphorylations before shutoff, is shown to contribute little to variability suppression.

We also find that, in addition to changed biochemistry, the geometry of the ROS might be important for the light response in mutant mice.

## Results

The technical aspects of the mathematical model are presented in **Methods**. Here we illustrate the main links between statistics, biochemistry and geometry. Label by the integer 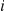 the state at which activated rhodopsin 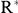 has acquired 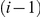 phosphates. Thus for example if 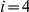 then 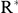 has acquired 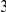 phosphates. Then either 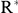 can acquire a further phosphate at a rate 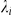 (determined by RK phosphorylation rate), or it can be quenched by Arr at a rate 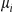 (determined by Arr on-rate). While in the 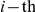 state, 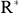 activates G protein with catalytic activity 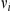. Finally 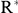 remains in the 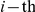 state a random sojourn time 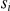, of mean 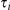. This is a typical sequence of Bernoulli trials whose statistical description by a Continuous Time Markov Chain (CTMC) is well known and standard [2], [14]–[16].

The main point of the model is in introducing a theoretical scheme that identifies the parameters of each of these steps in terms of their biochemical role. It turns out that WT responses alone are not sufficient to identify the parameters 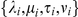. They are identified using recent experimental data obtained in genetically modified mice ([17]–[20]).

When these parameters are identified, the CTMC translates the deactivation cascade into the probabilities 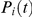 for rhodopsin to be in the 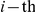 state at time 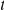. The output of the activation/deactivation cascade, computed by this CTMC scheme, and measured in terms of activated effector 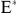, is then used as input in the spatio-temporal model introduced in [8]–[11]. The latter describes the dynamics of the second messengers cGMP and 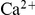 in the cytoplasm of the ROS, and the generation of photocurrent 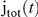 flowing through the cell membrane of the ROS, as a function of time 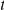. These two modules, so interfaced, provide a systems approach to phototransduction by mathematically separating, and then blending, the random events of the activation cascade occurring on a disk, the diffusion of second messengers in the cytoplasm, and current suppression on the outer shell.

**Figure 1:**
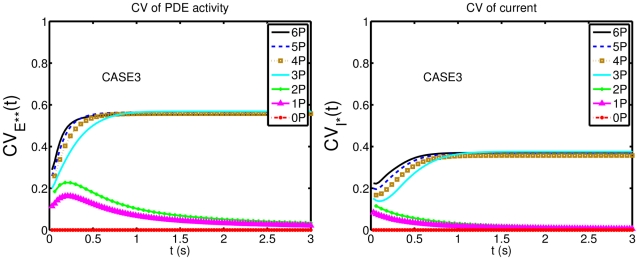
Comparing the CVs of the total activated effectors 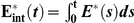 at time 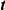 with the CVs of the total relative charge 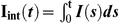 up to time 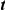 **.** All simulations assume both the sojourn time and the number of 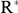 shutoff steps as random (Case 3 of Test Cases). The CVs of both 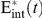 and 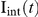 stabilize asymptotically for three or more phosphorylation sites (3P–6P). A CV of about 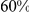 for 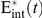 at times past the peak time is reduced to a CV of about 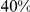 for the corresponding photocurrent 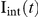. This points to an intrinsic variability reduction effect of the diffusion part of the process.

The variability of the effector 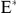 is described by the following functionals:
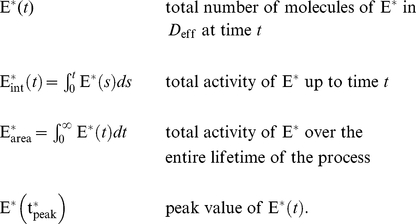
(1)The last two are scalar quantities and their CV is reported in Table 1. The first two are functions of time. The CV of the second, as a function of time is reported in Figure 1 (left). The natural variable functionals of the photocurrent are
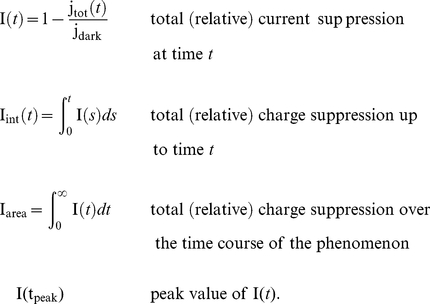
(2)While the last one is the value of the first at peak time, we have listed it separately since it is frequently reported in the literature [5], [6], [21]. 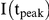 is a scalar quantity and its CV is tabulated in Table 1. The first two are functions of time. The CV of the second is graphed as a function of 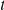 in Figure 1 (right). The quantity 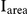 is the total relative charge produced over the entire time course of the phenomenon following isomerization by a single photon and is referred to as the SPR area [1], [2], [4], [22].

**Table 1:** Coefficients of variation, 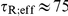 ms.

Sites		0P	1P	2P	3P	4P	5P	6P(WT)
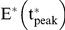	**Case1**	0.00	0.12	0.21	0.35	0.40	0.44	0.46
	**Case2**	0.00	0.00	0.00	0.00	0.00	0.00	0.00
	**Case3**	0.00	0.12	0.21	0.35	0.40	0.43	0.45
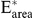	**Case1**	0.00	0.02	0.03	0.57	0.56	0.57	0.57
	**Case2**	0.00	0.00	0.00	0.00	0.02	0.02	0.03
	**Case3**	0.00	0.02	0.03	0.57	0.56	0.56	0.55
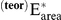	**Case1**	-	-	-	0.56	0.54	0.52	0.51
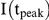	**Case1**	0.00	0.04	0.07	0.16	0.23	0.27	0.30
	**Case2**	0.00	0.00	0.00	0.00	0.00	0.01	0.01
	**Case3**	0.00	0.04	0.07	0.17	0.22	0.26	0.29
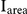	**Case1**	0.00	0.01	0.01	0.37	0.36	0.37	0.38
	**Case2**	0.00	0.00	0.00	0.00	0.01	0.02	0.02
	**Case3**	0.00	0.01	0.01	0.38	0.36	0.37	0.37

CV (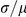) calculated for a 3 s simulation and 5000 trials for each of **Case 1**: Fixed number of steps to 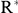 shutoff and random sojourn times 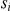; **Case 2**: Fixed sojourn times 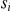 and random number of steps to 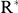; **Case 3**: Both sojourn times 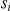 and 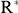 shutoff steps are random. The parameters 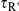 and 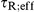 and their equivalence for WT mouse are discussed in the section 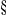 **Parameters**. The theoretical values of 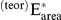 are reported for 3-6P as the theoretical formula of Eq:3-Eq:4 is valid only for these cases.

### Simulating the SPR in Transgenic Mice

Deactivation of rhodopsin with one or several mutant phosphorylation sites, can be simulated by suitable choices of the sequences 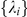 and 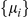 as indicated in the section of numerical procedures and methods.

**Figure 2:**
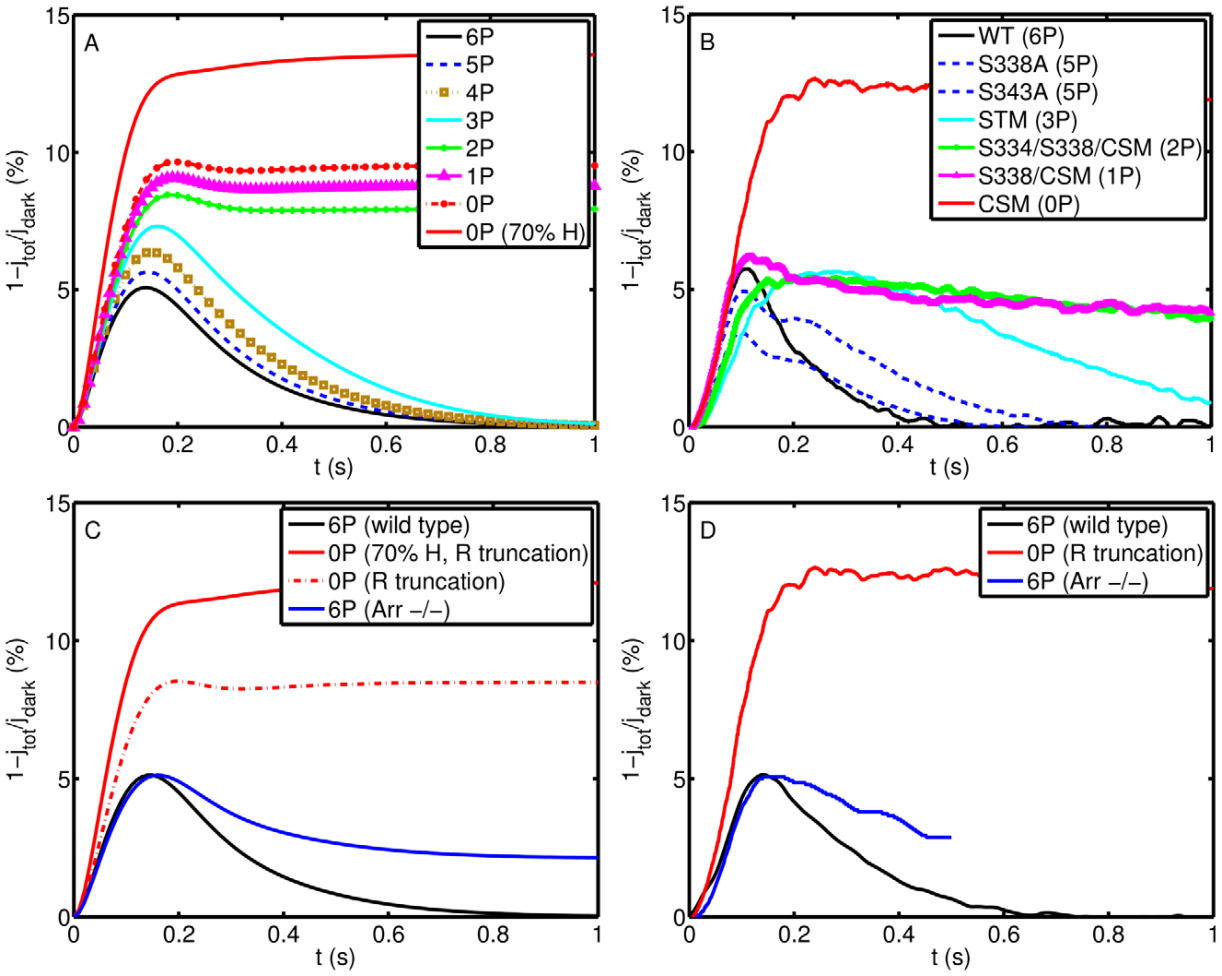
Simulations SPR for mutant phosphorylation sites of 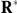, or with Arr knockout. **Panel A**: Simulated SPRs for rhodopsin with a number 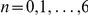 of available phosphorylation sites (thus 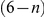 sites are mutant); **Panel B**: Reproduction of data from [3] showing SPRs from mutant mice with different phosphorylation sites. CSM: completely substituted mutant (0P); STM: serine triple mutant (3P); S338A: mutant lacking S338 (5P); S343A: mutant lacking residue S343 (5P); S338/CSM: one site (S338) was restored in the CSM (1P); S334/S338/CSM: two sites (S334 and S338) were restored in the CSM (2P); Mutant rhodopsins bearing zero, one (S338), or two (S334/S338) phosphorylation sites generated single-photon responses with greatly prolonged durations. Responses from rods expressing mutant rhodopsins bearing more than two phosphorylation sites declined along smooth, reproducible time courses; the rate of recovery increased with increasing numbers of phosphorylation sites; **Panel C**: Simulated SPRs with no phosphorylation site (0P), lacking arrestin (–/–), and wild type (WT); **Panel D**: Reproduction of the SPRs from rod with C terminal truncation, lacking arrestin (–/–), and wild type (+/+) [24] rescaled to exhibit the same proportional amplitude as the wild type SPR. The simulated curves were rescaled accordingly. With arrestin absent, the flash response displayed a rapid partial recovery followed by a prolonged final phase. This behavior indicates that an arrestin-independent mechanism initiates the quench of rhodopsin's catalytic activity and that arrestin completes the quench. Analogous simulations for the faster dynamics 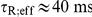 and 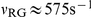 are in Figure S2 of the supplementary material.

Mutant mouse rhodopsins bearing fewer than 6 phosphorylation sites generate SPRs of significantly extended durations (Figure 2A). The rate of recovery increases with increasing numbers of phosphorylation sites (Figure 2A), in qualitative and quantitative agreement with the experimental results of [3] (Figure 2B).

Inactivation of all rhodopsin phosphorylation sites is realized by either mutation of all six serines and threonines to alanines [1], [3], or rhodopsin kinase knockout [23]. The corresponding SPRs are similar, exhibiting larger amplitude and longer duration than WT (Figure 2B,D for (0P)).

A prolonged SPR in mutant mouse rods lacking arrestin is reported in [24] (Figure 2D). This is realized by setting 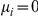 for all 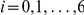 in the model. The activated rhodopsin gets phosphorylated until all six sites are occupied. Its activity is reduced with increased phosphorylations, and kept fixed after the last phosphorylation for the remainder of the process. The remaining activity yields a response with an asymptotic tail at almost half of its peak value. The initial fall of the response is triggered by phosphorylation. The simulations are shown by (–/–) in Figure 2C, and are qualitatively and quantitatively in agreement with the experimental studies of [24] (Figure 2D).

In Table 2 we report the simulated characteristics of SPRs from WT rods and those expressing rhodopsin mutants. By increasing the number of phosphorylation sites, the peaks of the current response 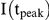 decrease; the time to peak 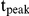 decreases; and the SPR area 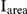 decreases significantly. For mutants that exhibit very slow recovery (0P, 1P, 2P) the corresponding 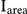 is large because the current remains high for an extended period of time. The value of 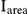 has been computed by integrating the photocurrent over the time of simulation (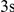).

**Table 2:** Characteristics of SPRs, 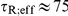 ms and 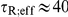 ms.

	Rhodopsin	0P	1P	2P	3P	4P	5P	6P(WT)
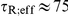 **ms**	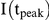	**8.54**	**8.02**	**7.50**	**6.83**	**6.19**	**5.62**	**5.13**
	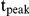 (s)	0.20	0.19	0.19	0.17	0.16	0.15	0.14
	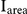 (s)	24.81	22.35	19.60	2.89	2.11	1.74	1.51
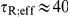 **ms**	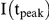	**9.66**	**9.08**	**8.47**	**7.32**	**6.38**	**5.65**	**5.08**
	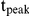 (s)	0.19	0.19	0.19	0.16	0.15	0.14	0.14
	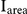 (s)	27.86	25.74	23.34	2.73	2.07	1.74	1.52

Characteristics of SPRs from Wild Type and Rhodopsin Mutant Rods from 3 s simulations for the dynamics 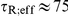
ms and 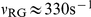 and 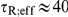
ms and 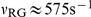. The parameters 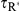 and 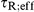 and their equivalence are discussed in 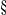 **Parameters**.

When only one phosphorylation site was mutated, the SPR was almost like that of WT but recovery was slightly slower. Consistent with this slower recovery, the SPR area 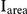 of the response of rhodopsin with five phosphorylation sites (5P) was about 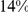 larger than those for wild type. Taken together, these results are consistent with the experimental observations of [3] and the notion that normal kinetics of 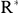 deactivation requires the presence of all six phosphorylation sites.

**Figure 3:**
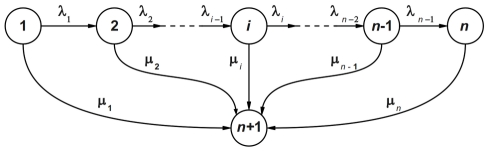
State diagram of CTMC model for rhodopsin deactivation. States 1 to n are active states and state n+1 is the inactive state. The phosphorylation rates and arrestin binding rates are denoted respectively by 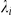 and 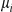.

We finally comment on the largest rising curves coded in red in Figure 2B and D. Various experimental studies [3], [23] show that the response amplitude for the case (0P) is roughly twice as large as the response for the case (1P). In [3] the case (0P), is realized by CSM, and in [23] by RK knockout. In both cases all phosphorylation sites are removed or made inoperative, and both cases exhibit the double amplitude response, suggesting a common mechanism. This issue is not discussed in the indicated papers and we are not aware of an explanation or hypothesis for a possible biochemical mechanism. However, Figure 2 of [3] shows that the ROS in CSM mice were about 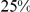 shorter than WT. Geometrical changes due to genetic manipulations are also discussed in [23] (page 3720), and [24], (Figure 3d, page 506). We repeated the simulations with a ROS whose height 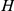 was reduced by 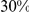, while all the remaining parameters were kept fixed. In particular, the number of channels was kept fixed, thereby increasing their density. Since the response is localized close to the activation site [10], [11], the augmented channel density yields a larger response. The resulting simulation is reported in Figure 2A for (0P*) as the largest amplitude (red curve). While the agreement with corresponding experimental curve in Figure 2B is striking, at this point we refrain from suggesting that this as the only functional mechanism.

### Variability

The CTMC model permits one to test independently the effects of the random components of the variability on the response. For example one can separate the effects of the randomness of the sojourn time from the randomness of number of shutoff steps. To achieve this, we performed the following sets of simulations:

- **Case 1**. Fix the number of steps to 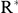 shutoff at that integer closest to its mean 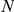, and let 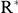 have random sojourn time 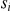 at the corresponding state. The random numbers 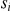 are generated according to their exponential distribution with mean 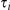.
- **Case 2**. Fix the sojourn times of 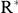 at their mean 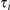 and let 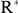 be shut off in 
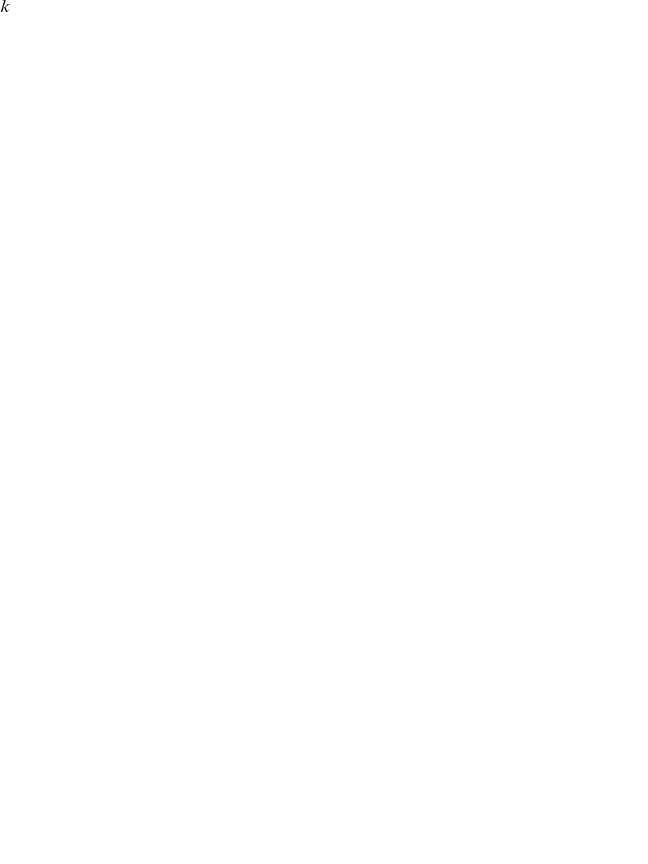
 random steps. The random number 
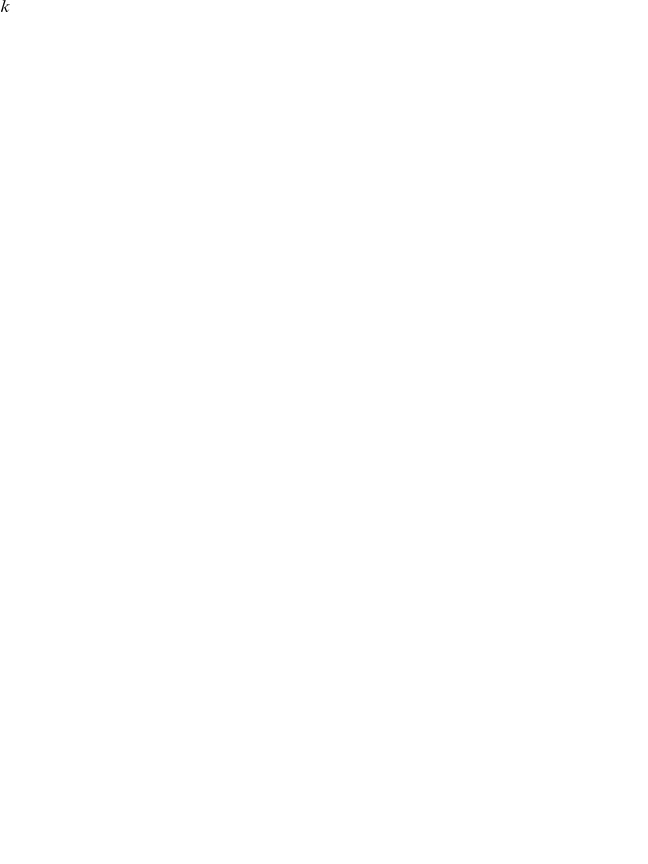
 of 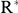 shutoff steps is generated by a series of Bernoulli trails, in which the probability of phosphorylation is 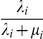 and the probability of Arr binding is 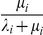. Thus the mean 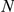 of the random number 
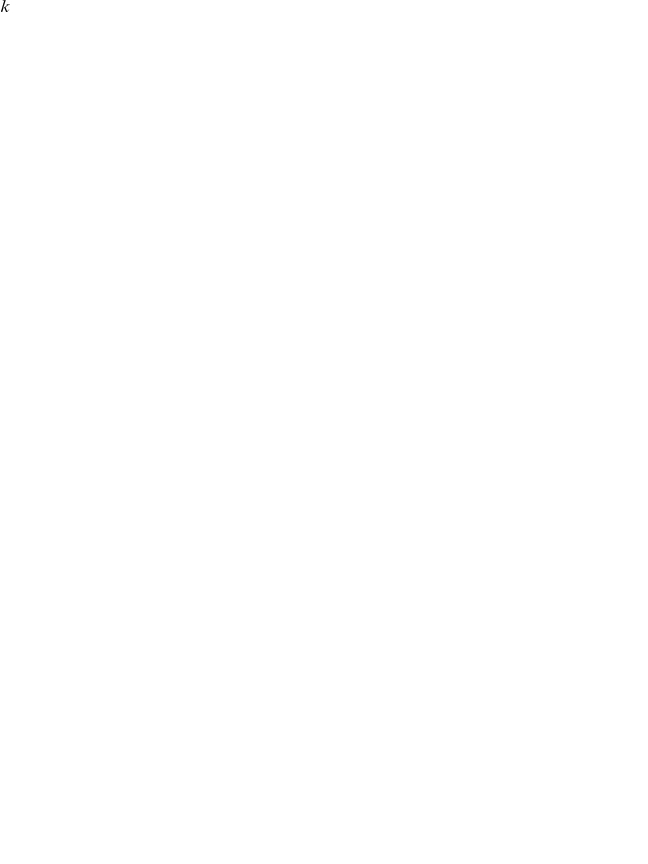
 is computed from Eq:10–Eq:11.
- **Case 3**. Both sojourn time 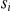 and the number of shutoff steps 
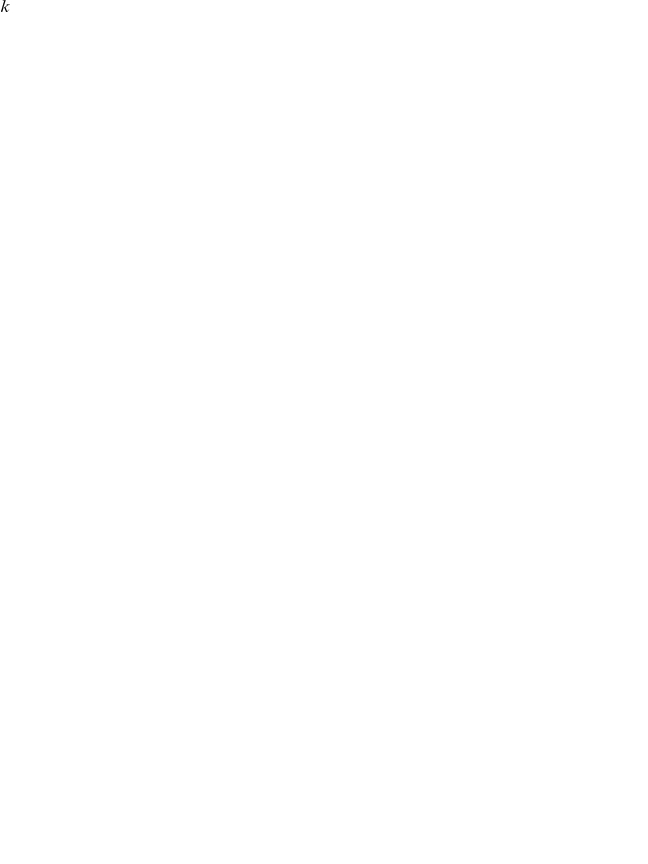
 are random. This is the biologically realistic case, although the previous cases extract the effect of the randomness of each component on the variability of the response.

Stochastic simulations are effected for WT and each of the knock-out cases of COOH-terminal truncations [24], [25] and RK knockout [1], [3]. After about 5,000 numerical simulations, up to 3 s, mean, standard deviation and CV are computed for effector and normalized current suppression. Further technical details are in **Methods**.

#### Variability of 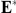


The first two lines of Table 1 report the CV of the scalar quantities 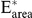, and 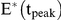 defined in Eq:1, and for 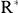 bearing 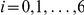 phosphorylation sites. The first result is that the CV for **Case 2** is negligible (computationally up to 2 decimal points). This indicates that the randomness of the number of 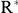 shutoff steps does not significantly contribute to the CV of 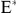. The second result is that the CVs produced by **Case 1**, to which only the randomness of sojourn time of 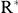 contributes, are roughly the same as those of **Case 3**, where all components are allowed to be random. It appears from the table that the randomness of the sojourn times of 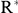 in its phosphorylated states is largely responsible for the CV of 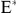 in this model.

For mutant 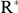 with zero phosphorylation sites (0P), the CV of any of these quantities is zero in all cases. Since 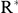 could neither be phosphorylated nor be bound by Arr (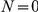), it remains in state 1 indefinitely and the process has no random components, within the time scale of the simulation. On a longer time scale, eventually active metarhodopsin II releases bound all-trans-retinal and decays to opsin, losing most of its ability to activate transducin. It is not surprising that 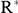 with deactivation deficit leads to a highly reproducible SPR in the very first few seconds (3 s in our simulations), as no inactivation occurs.

The observations in [1] (see Figure 3, Panel F of [1]), indicate that the SPRs generated by unphosphorylated 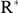 are highly reproducible within the very first few seconds (about 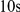). Later shutoff of unphosphorylated 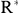 is believed to be due to thermal decay of 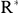 to opsin [12]. Here we are interested in the deactivation of 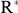 within the time scale of normal SPR (3 s in our simulations) and the effects that could be involved beyond this time period are not considered.

For mutant 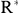 with one phosphorylation site (1P, 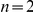), the CV of any one of the variability functionals in Eq:1–Eq:2 is very small. Such a mutant 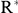 could be phosphorylated to one level, but it could not be shut off by Arr binding since mono-phosphorylated 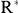 has the same low Arr binding levels as unphosphorylated 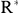 (see the discussion in **Methods** and [20]). The randomness of one extra level of phosphorylation causes a noticeable increase in uncertainty as measured by the CVs. From Eq:7–Eq:11 one computes 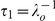 and 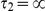. Therefore 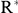 remains in the unphosphorylated state 1, for a random sojourn time 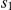 of mean 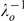; then it transitions to state 2 by acquiring a phosphate and it remains indefinitely in that state. The only randomness is due to the sojourn time 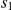, which affects the CV of 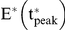. Since 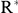 is never turned off (within the 3 s time frame used here), the functional 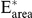, is uniformly large for all trials, and therefore it exhibits negligible variability.

Compared with the CV of 1P, the mutant 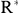 with two phosphorylation sites (2P, 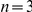) exhibits a larger CV for any of the variability functionals, the increase in uncertainty being due to the second phosphorylation site. The only randomness of the process is due to sojourn times 
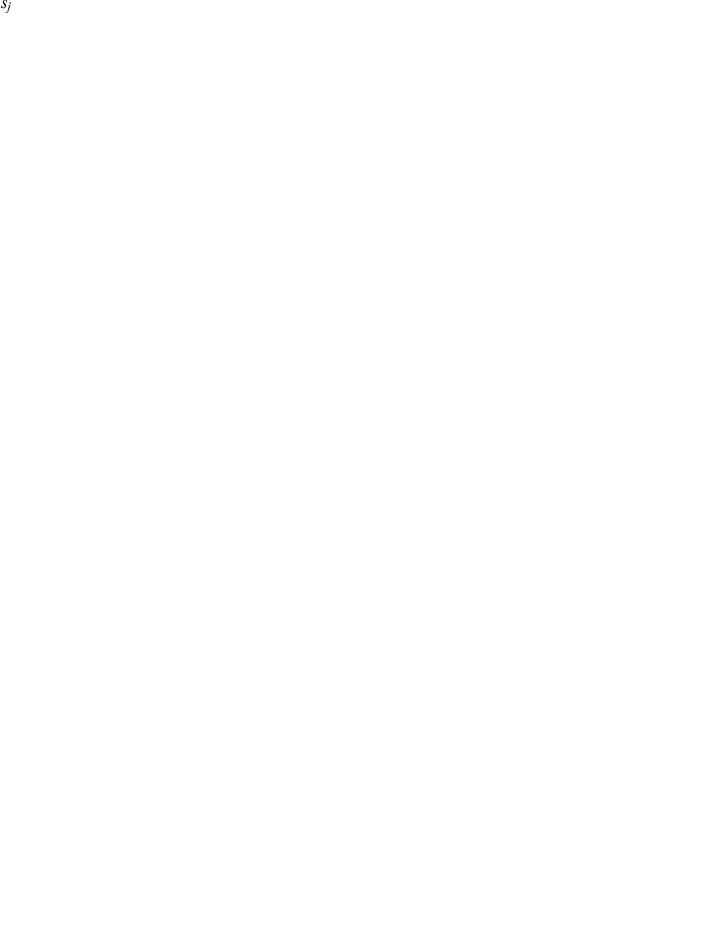
 of means 
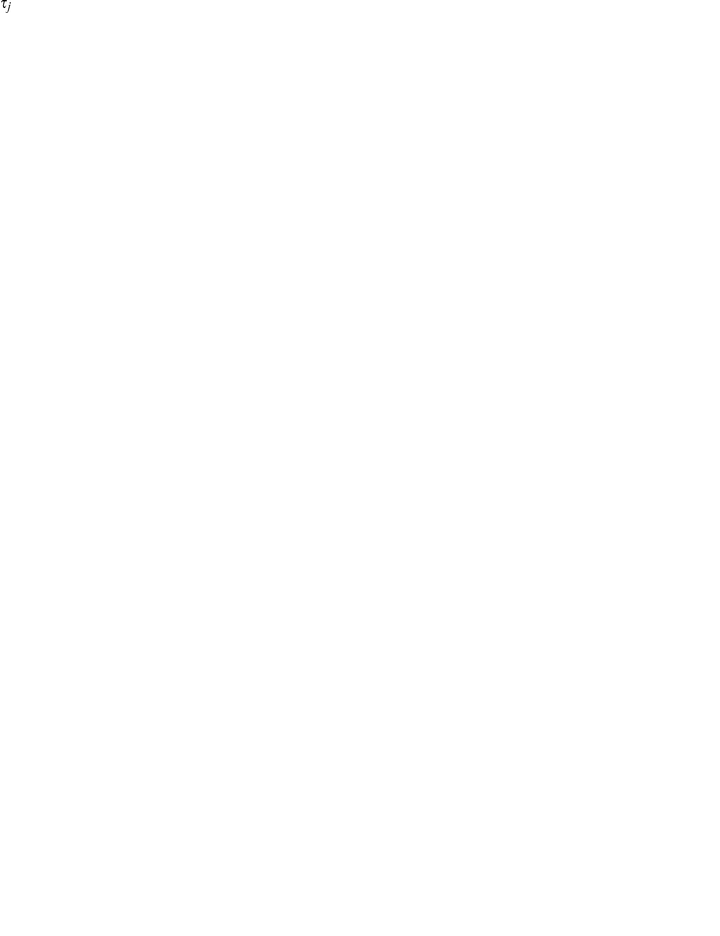
, as the number of possible steps (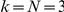) is not random. In the case of 2P the uncertainty of 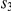 is compounded, with respect to the case 1P, by the uncertainty of the random sojourn times 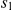 and 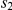, although their mean is smaller. Accordingly all functionals exhibit larger variability. Also for the case 2P shutoff does not occur since 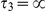 (from Eq:7–Eq:9). Therefore, for the cases 0P, 1P and 2P, the CVs of the functionals 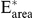 and 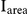 reported in Table 1 is not due to variations caused by inactivation, as the latter, theoretically, never occurs. In reality, inactivation does occur, although by different mechanisms, for example thermal decay to opsin, on a much larger time scale.

As the number of available phosphorylation sites increases (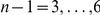), one might expect that the uncertainty of the sojourn times 
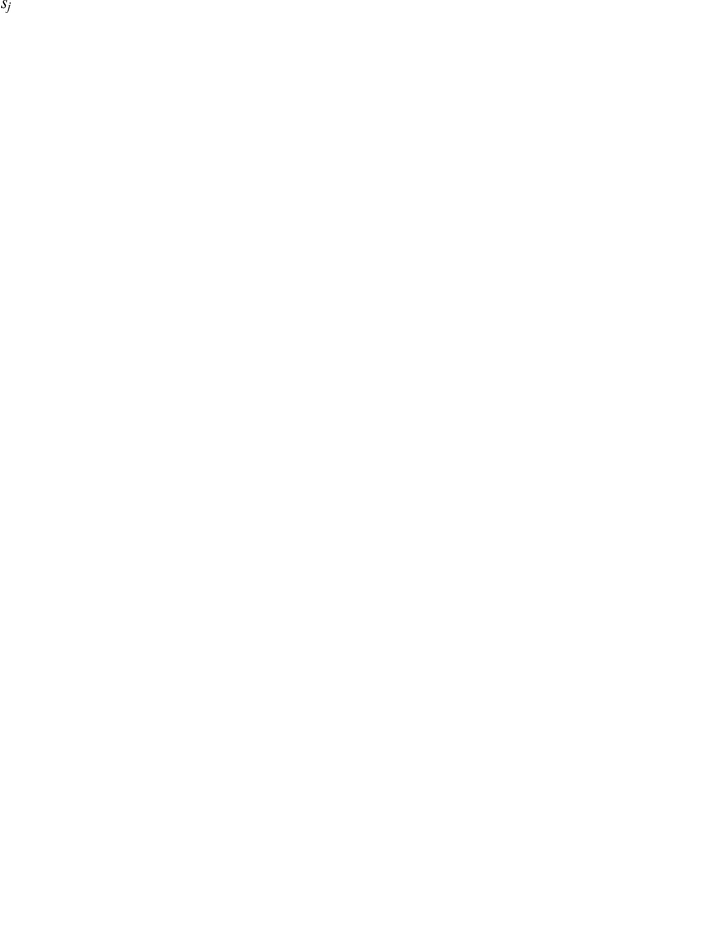
, be compounded by the randomness of the number of steps 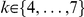 to 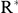 shutoff. However Table 1 shows no significant difference in the CV of all functionals, between Case 1, where the number of steps to 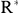 shutoff is kept fixed to its mean 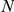, and Case 3, where all components are permitted to be random. This suggests that the behavior of the various CVs reflects the randomness of the sojourn times.

For 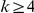 fixed at its mean 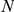 (**Case 1**), the CV of 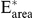 is computed by the explicit formula [8]

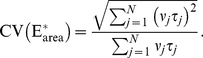
(3)This formula is valid provided
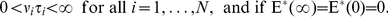
(4)The latter condition stipulates that the system returns to its original dark state after a sufficiently large time. Therefore this formula holds true only for the cases 3P-6P.

For **Case 1**, with the number of steps to shutoff fixed at the closest integer to the mean 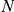, we have computed explicitly the sequences 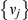 and 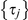 from Eq:7–Eq:9 and have computed the corresponding CV from formula Eq:3. These theoretical CVs are reported in line 7 of Table 1 and show a reasonably good agreement with the simulated values of CV(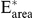).

In Figure 1 (left), we report the graphs of the CV for 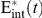 as function of time, only for Case 3. Indeed, this is the biologically realistic case, where all the components of the phenomenon are permitted to be random. This variability functional is defined in Eq:1. Similarly as observed in the context of Table 1, the CV for 0P is negligible and the CV of 1P and 2P are relatively small.

The CV of 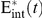, for wild type (6P), stabilizes from 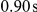 with a value of 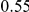, and the CV of the same functional for 3P, stabilizes from 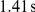 with a value of 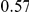. By increasing the number of phosphorylation sites from 3P to 6P, the stabilized CVs of the functional 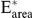 decrease (Table 1), and the time at which the CVs begins to stabilize decreases.

The functional 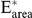 compounds the variability of the process at all times, up to recovery, and therefore its CV is expected to be larger than the CV of 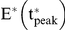.

#### Variability of the photocurrent

In the last two rows of Table 1 we have reported the CV of the scalar quantities 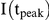 and 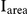, defined in Eq:2, for each of the **Test Cases 1,2,3**, and for a 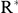 bearing 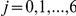 phosphorylation sites. The results exhibit a pattern similar to the CVs of 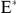 and 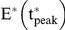 although at considerably lower values of CV. A CV of about 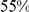 for 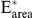 is reduced to a CV of about 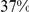 for the corresponding photocurrent 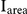. Thus the diffusion part of the process acts as variability suppressor, in agreement with the results of [8].

The simulations show that CV of 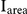 is essentially constant with respect to the number of available phosphorylation sites 3–6.

Figure 1 (right) reports the CV of the total relative charge 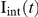 produced up to time 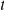, for the physically realistic Case 3, where all random components are present. The results exhibit a pattern similar to those in the left panel of the CV for 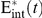 although, again, at considerably lower values of CV. The CV of 0P is zero and the CV of 1P and 2P is relatively small. For 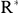 with three or more phosphorylation sites, the CV increases with increasing phosphorylation sites, at the early times of the activation. Thereafter, the CVs for different number of phosphorylation sites tends to stabilize with stabilization time inversely proportional to the number of available sites, i.e., the more sites 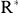 has, the faster CV stabilizes.

## Discussion

Variability of the photoresponse hinges on a coordinated system behavior of several components. The main two modules are the activation/deactivation part and the transduction part of the cascade. The latter, given its input, is essentially deterministic as it involves the diffusion of the second messengers cGMP and 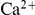 in the cytoplasm and a subsequent current drop through the closure of the cGMP -gated channels. The former is essentially stochastic as it involves the biochemistry of rhodopsin shutoff, which occurs in several random steps. An understanding of the process hinges upon teasing apart all these components, analyzing them separately and blending them together into a system behavior. This point of view began in [8], by separating the role of the transduction from that of the activation/deactivation. This separation was made possible by a mathematical model capable of distinguishing the biochemistry of 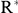 shutoff, from the functional role of the transduction [8], [9], [11]. A surprising finding was that, while 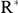 shutoff is responsible for the variability of the photoresponse, the diffusion of the second messengers acts as a variability suppressor.

Here we have further separated the various steps of the deactivation cascade by (a) prescribing a probabilistic mechanism (CTMC) by which the system selects its random states, and (b) by interrogating the known biochemistry to trace patterns and parameters.

It is not sufficient to determine these parameters unambiguously using WT mice. Experimental information from some mutant and knock-out animals is needed. Specifically, the choice of the catalytic activities 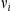 by formula Eq:6, while based on known biochemistry [26], hinges upon the basic parameter 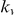, which in turn is determined by the biochemistry of the cascade in mutant mouse (section on parameters in **Methods**). The same holds true for the transition parameters 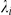, given by formula Eq:7 and depending upon the parameter 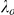. Thus, a first remark is that our approach, while mathematical and computational, parallels the biology; that is, information is extracted in a complementary way from the data on genetically modified as well as WT animals. Next the model populated by the indicated parameters is validated against WT and mutant responses as in Figure 2. The model has a deterministic component, and a stochastic component. The first regards the transduction part of the cascade, which is geometry dependent, and deterministic, being based on the diffusion of the second messengers cGMP and 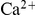 in the cytoplasm.

Importantly, this model permits one to test the response against geometrical variations of the ROS. The response in mice expressing CSM or RK knock out is rather unusual, exhibiting a double amplitude with respect to WT [3], [23]. An examination of the immunofluorescence micrographs in Figure 2 of [3], suggests that the length of ROS in CSM mice is reduced by about 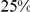 relative to WT. Geometrical modifications presumably due to genetic manipulations are also discussed in [23]. Keeping the same stochastic biochemical scheme and changing the length of the ROS, the model reproduced the double-amplitude phenomenon described in [3], [23] (Figure 2 A,B), suggesting that the modified geometry of mutant ROS, might contribute, along with the changed biochemistry, to this phenomenon. This results, along with a recent study of rod signaling in mice expressing supra-physiological levels of rhodopsin ([27]), emphasize the importance of investigating the ROS geometry in genetically modified mouse lines. Our analysis shows that the changes in ROS length, which were analyzed in very few mouse lines, can have dramatic effects on photoresponse.

The stochastic component permits one to single out those parts of the activation/deactivation cascade that dominantly contribute to the variability of the response. The main result is that variability is largely generated by the randomness of the sojourn times of 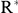 in its phosphorylation states. The prevailing point of view is that the activation cascade is responsible for the variability, although in a non quantified way, and that deactivation of 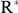 is responsible for variability suppression, and further, the larger the number of decay steps of 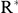, the more stable the photoresponse [1], [3]–[7]. This view was expressed in [1], where mice expressing rhodopsin with 0,1,2,5, and 6 phosphorylation sites were used. The analysis presented in [1] has some inconsistencies. Although the experimental points seem to be best fitted by a straight line (Figure 1 of [1]) the authors describe them by 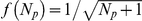, with 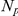 being the number of available phosphorylation sites. The lines with 3 and 4 phosphorylation sites, which would have allowed to discriminate between these functions, were not analyzed in [1]. In addition, by comparing the CV of mice with 0,1, and 2 sites, which do not demonstrate rapid recovery ([4]), with those having 5 or 6 (WT) sites that recover with essentially the same fast rate, the authors inappropriately lump together two disparate phenomena. In the latter case, normal two-step rhodopsin inactivation by RK phosphorylation and arrestin binding is fully operative, whereas in the former rhodopsin is inactivated by stochastic thermal decay taking place on a much longer time course. The idea that multiple inactivation steps are necessary to suppress variability was recently expressed in [2], where the authors conclusions were largely based on two assumptions. The first is that 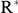 activity is nearly equally distributed among the deactivation steps. The second is that in Ames' solution, that yields much greater and longer-lasting SPR than Locke's ([2], [28]), rhodopsin inactivation is rate-limiting and dominates the recovery kinetics. The biochemical scheme we propose argues against the first assumption, on experimental grounds (see a discussion below and **On the Parameters** 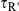 **and** 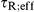). The second assumption has been recently questioned in [28], where the authors showed that RGS9 overexpression similarly accelerates the recovery measures in Locke's and Ames' solutions, indicating that transducin inactivation is rate limiting in both cases. Additional issues with data analysis of [1] were discussed in [28]. Thus, no compelling experimental evidence that the number of inactivation steps reduces variability can be found in the literature.

Our results offer a different perspective; demonstrating that variability is generated by the randomness of the sojourn times of 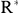 in its phosphorylated states, and that increasing the number of these states does not lead to variability suppression.

The number of steps to deactivation does not coincide with the number of available phosphorylation sites. The experimental studies of [20] suggest that one phosphorylation is not sufficient for Arr binding, and the probability of quenching becomes large after 3 phosphorylations. Specifically 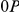 corresponds to 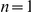 by which Eq:7–Eq:8 give 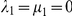 and hence 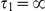 by Eq:9. Thus, the system remains indefinitely activated (in reality it is stochastically inactivated by the thermal decay of rhodopsin, which is too slow to be captured by 3 s simulations used here). The case 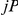 for 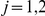 corresponds to 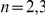 respectively and one computes 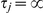 from Eq:9 and hence 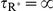 from Eq:12; the system goes through 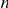 steps and then remains “indefinitely” active (see above about thermal decay). Thus the CV of 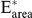 and 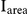 in Table 1 and Table S1 in the supplementary material, are not due to variations caused by 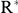 shutoff by Arr binding. The first case when 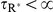 and deactivation is possible, is the case 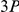 corresponding to 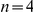 reported in Eq:13. Going from 3P to 6P, the 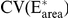 and the 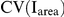 remain essentially the same.

To illustrate the rationale of our main results, consider mutant rhodopsin with only 3 available phosphorylation sites. Since 3 phosphates are needed for Arr binding [20], no randomness is present in the deactivation process, if randomness is only measured in terms of steps to shutoff. This suggest that the source of variability is in other components of the process. Table 1 indeed shows that if the sojourn times of 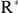 in each of its phosphorylation states are taken to be deterministic (Case 2), then the variability of the photoresponse is negligible. If on the other hand such sojourn times are permitted to be random, then the variability rises to experimentally observed levels (Figure 1), both for WT and genetically modified 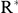 with 3-5P. This should not be interpreted, however, as though the reproducibility decreases as the number 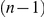 of available phosphorylation sites increases. We stress that increasing 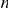 does not necessarily mean that the mean number 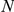 to 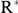 shutoff increases. The latter depends on the biochemistry of the process via Eq:10–Eq:11. Likewise the expected average 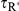 of the random lifetime of 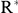 is generated by the biochemistry in Eq:7–Eq:12 and 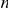; in particular it is different for different genetically modified mice (0P,1P,etc.). The lifetime of 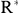 is randomly chosen by the biochemistry in each of its random trials.

For WT mouse, and only in this case, the expected lifetime 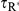 of 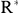, as defined by formula Eq:9–Eq:12, coincides with the experimentally measured, effective average lifetime 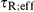. In [29] it is reported 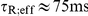 as an upper limit, whereas several recent studies [28], [30], [31] suggest that 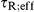 might be as low as 40 ms (see 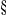 **Parameters**).

Therefore, we performed all simulations for both values, which yielded very similar CVs, both functionally and numerically (Figure 1 and Tables 2–3, and Figure S1 and Tables S1,S3, in the supplementary material). These similarities suggest that reproducibility is independent of the actual value of 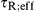 and depends only on the functional, sequence of the deactivation cascade, as predicted by our biochemical scheme (Eq:9– Eq:12). Further remarks on these two parameters and corresponding CVs are in 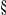 **On the Parameters 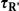 and 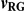
**.

**Table 3:** The sequences 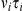 for the dynamics of 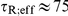
ms and 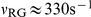.

**6P (WT)**	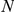	4.45						
	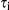	15.87	19.05	23.81	10.93	12.35	14.18	16.67
	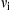	330.00	200.16	121.40	73.63	44.66	27.09	16.43
	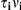	5.24	3.81	2.89	0.80	0.55	0.38	0.27
**5P**	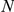	4.30						
	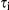	19.05	23.81	31.75	12.35	14.18	16.67	
	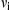	330.00	200.16	121.40	73.63	44.66	27.09	
	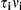	6.29	4.77	3.85	0.91	0.63	0.45	
**4P**	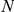	4.15						
	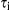	23.81	31.75	47.62	14.18	16.67		
	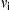	330.00	200.16	121.40	73.63	44.66		
	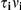	7.86	6.35	5.78	1.04	0.74		
**3P**	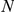	4						
	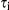	31.75	47.62	95.24	16.67			
	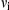	330.00	200.16	121.40	73.63			
	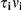	10.48	9.53	11.56	1.23			
**2P**	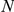	3						
	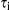	47.62	95.24	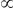				
	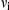	330.00	200.16	121.40				
	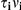	15.71	19.06	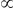				
**1P**	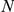	2						
	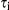	95.24	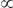					
	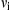	330.00	200.16					
	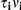	31.43	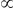					
**0P**	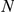	1						
	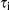	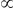						
	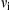	330.00						
	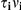	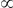						

The sequences 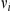 (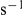), 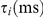 and the average number 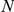 of steps to shutoff of 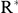, for WT and mutant mice, computed from Eq:9–Eq:11. Computation for the dynamics of 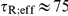
ms and 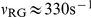. The parameters 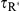 and 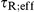 and their equivalence are discussed in 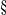 **Parameters**.

We stress that the model includes only the deactivation mechanism due to Arr binding and does not include 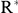 inactivation due to other causes such as thermal decay to opsin occurring over a time course of 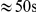 ([13]).

In [1] the 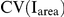 is computed over a time course of over 15 s, which is beyond the time course 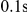 of 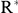 inactivation. According to our scheme, based on direct biochemical measurements of arrestin binding to separated rhodopsin species with different numbers of attached phosphates ([20]), cases 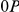, 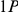 and 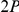 do not permit shutoff by Arr binding and 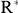 remains active much longer than the 3 s of our simulations. Thus the CV due to 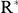 deactivation reflects its thermal decay to opsin ([12]). In this case, shutoff is an abrupt 1-step process, implying, by Poisson statistic, CV = 1. This is essentially what is reported in [1]. For the cases 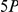 and 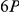, although the experiments of [1] are carried over a time course of 15 s the 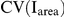 is essentially due to shutoff by arrestin binding, which occurs within a time course of 0.1 s, whereas decay to opsin is much slower. Considering the slow rate of thermal inactivation of rhodopsin, the probability of thermal decay within the first 0.1 s is negligible relative to the probability of decay due to Arr binding. Accordingly, the 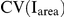 reported in [1] for 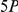 and 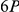 is similar, as we find. The crucial cases 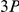 and 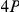 were not measured in [1].

The CTMC scheme we propose here differs from the Poisson statistics used in [1], [2], where the CV of 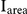 is claimed to be proportional to 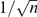. It should be noted that the number of available sites does not coincide with the average number of steps to shutoff and that each step weighs differently in the deactivation process, due to its biochemical history.

We return briefly to the explicit, theoretical formula Eq:3, valid under the assumptions of Eq:4, and hence for the cases 3-6P. We have already remarked that its theoretical values (for **Case 1**) are in agreement with our simulations (lines 2 and 3 of Table 1). If one would artificially concoct a biochemistry by which all the products 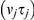 are the same for all 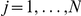, then formula Eq:3 would give 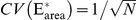. This occurrence might suggest that the CV of the photoresponse decreases as the reciprocal of the square root of the number 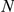 of steps to shutoff. A calculation from Eq:7–Eq:11, in agreement with known biochemistry ([20]), shows that the products 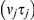 are not constant (Table 3). In addition, even if this were the case, the variability of the photocurrent is very different from that of 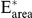, as the relation between these functionals is highly non-linear [8], [11].

A further examination of Table 3 for 3-6P, shows that in all cases (WT or mutant), only the first few steps contribute significantly to the total activity 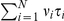; the remaining ones being negligible. In view of the theoretical formula Eq:3, this is further evidence that increasing the number of steps does not significantly decrease the CV(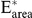).

In all cases (WT or mutant) we found that the diffusion of the second messengers cGMP and 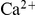 in the cytoplasm acts as the dominant variability suppressor, thereby confirming the results of [8] and extending the analysis to a variety of transgenic models.

These results are made possible by separating the activation/deactivation module from the transduction module. In addition, in the activation/deactivation module, one further separates the biochemical effects of each phosphorylation contributing to the responses, thereby allowing an examination of the role of the underlying biochemistry during 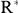 deactivation. Incorporating the sequence of biochemical steps, described in **Methods**, allowed us to recapitulate experimental results qualitatively and quantitatively (Figure 2). It is worth noting that with realistic biochemistry, where Arr acquires a high binding affinity after 3 phosphorylation steps [20], the number of inactivation steps actually involved in shutting down individual SPRs varies very little. Therefore the fact that this number contributes virtually nothing to SPR variability, is one of the mechanisms maintaining the reproducibility of SPR.

## Methods

### The Mathematical CTMC Model

The state diagram of the CTMC describing 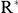 deactivation by Rhodopsin Kinase (RK) phosphorylating the C-terminal serines and threonines in rhodopsin, is shown in Figure 3 with circles and arrows denoting states and transitions respectively.

The states are labeled by the indices 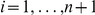, and the transitions between connected states are labeled by transition rates 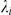 and 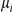. The 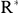 catalytic activity in its 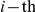 state is 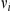. The number 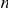 of phosphorylation levels is determined by the number 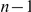 of phosphorylation sites of rhodopsin, which varies in different species. In mouse, rhodopsin has six phosphorylation sites [3]. State 1 is the non-phosphorylated level, representing newly activated rhodopsin with catalytic activity 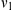; the state 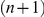 represents fully deactivated rhodopsin with catalytic activity 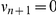; states 2 to 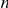 represent different phosphorylation levels, in which rhodopsin holds 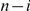 sites available for phosphorylation, with 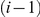 sites already phosphorylated, and has catalytic activity 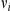. The states 1 to 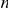 are active states and the state 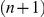 is the inactive state. Specifically for WT mouse, there are seven (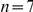) active states, including state 1 where 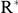 is active and not phosphorylated. Transitions between active states are governed by the phosphorylation rates 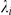. For notation consistency, we let 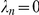. Transitions between active states and the inactive state are governed by the arrestin binding rates 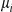. Arrestin binds with high affinity only to phosphorylated rhodopsin [20], [26], [32], [33], therefore, 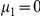.

A newly isomerized rhodopsin is in state 1. It undergoes a random number of phosphorylations before it transitions to the fully deactivated state 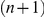. A rhodopsin with 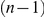 available phosphorylation sites could be phosphorylated at most 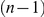 times to state 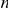. Generally, in state 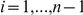, rhodopsin either interacts with rhodopsin kinase adding one more phosphate and transitions to the next phosphorylation level with a rate of 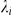, or it binds arrestin which quenches its catalytic activity, and transitions to the inactive state with a rate of 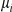. This process is a Bernoulli trial with the probability of a further phosphorylation given by 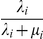 and the probability of arrestin binding given by 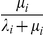. This statistical scheme permits one to model rhodopsin deactivation also in transgenic animals with different number of rhodopsin phosphorylation sites. For example, if 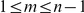 of the 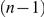 phosphorylation sites are mutated, we could set
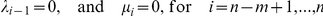
to reflect the effect of the mutation. It should be pointed out that, given 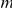 mutated sites, the model removes any 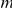 of the available sites with no discriminating criterion such as their ordering on the C-terminus. Although the phosphorylation of different sites in WT rhodopsin apparently proceeds in some order [34], the overall number of rhodopsin-attached phosphates, rather than their positions on the C-terminus, determines arrestin binding [3], [20], [35]. Accordingly, the model treats them as equal by attributing them the same biochemical function of holding a phosphate.

Let 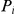 denote the probability that a single 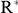 is in the state 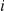. Then the mathematical description of the CTMC model shown in Figure 3 is [14], [15]

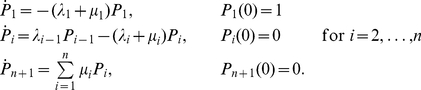
(5)Note that the integer 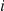 used to label the state of 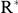 is one plus the corresponding level of phosphorylation, which is 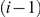. For example, the phosphorylation level 0 corresponds to state 1, and 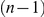 sites are phosphorylated in state 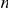. The sojourn time 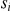 of 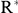 in state 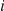, is taken as an exponentially distributed random variable with mean 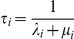. The sequences of the phosphorylation rates by RK 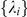, the activities of Arr 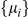, and the catalytic activities 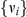, depend on the underlying biochemistry, and vary with phosphorylation levels [26], [36].

### The Sequence 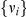 of Catalytic Activities of 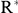


The catalytic activity 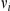 of 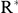 in the 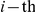 state is the production rate of activated G protein 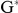 by 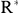. While 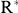 is active in each state 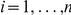, including the first unphosphorylated state (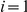), its activity decreases with increasing phosphorylation levels. The catalytic activity of rhodopsin with different numbers of attached phosphates was experimentally measured by Wilden [26]. In this study differentially phosphorylated rhodopsin species were actually separated, so the conclusions were based on direct measurements and did not involve untested assumptions. Although similar conclusions were later reached by Gibson et al. [32], these authors did not separate differentially phosphorylated rhodopsin species, using preparation with different average phosphorylation levels instead. Their calculations are based on the assumption of Poisson distribution of rhodopsin species with different number of phosphates ([32]). This does not appear realistic, considering the distribution determined by rhodopsin fractionation ([26], [33]). Therefore, our model assumes that the binding affinity of phosphorylated 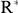 for 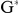 decreases exponentially with each added phosphate. Thus, based on data published by Wilden [26], we assume
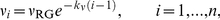
(6)where 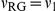 is the catalytic activity of 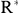 in its initial, unphosphorylated state, and 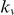 is positive. The value of 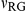 in Eq:6 has been extracted from the published data after an extensive consistency and sensitivity analysis ([37]). The parameters 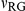 and 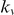 are linked and subject to mutual limitations. It has been shown that in arrestin knockout mice, the initial kinetics of single photon response deactivation closely resembles that of WT, whereas the later phase of deactivation is abrogated (Xu et al., 1997). Initial deactivation is attributable to rhodopsin phosphorylation, which is preserved in these animals. Then deactivation stops at about 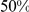 of the peak current suppression, and remains essentially steady thereafter. This level of current drop reflects the ability of fully phosphorylated mouse rhodopsin to activate transducin, corresponding to the catalytic activity 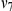 when all 6 sites are phosphorylated. Thus from Eq:6 one has 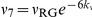. Mutual calibration of 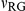 and 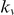 is discussed in the section on parameters. Here we stress that they are determined from experimental data for both WT and mutant mice, and not chosen by fitting.

### Phosphorylation Rates {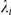} and Affinity of R-RK

While the explicit dependence of 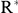-RK binding affinity and the 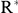 phosphorylation rates on the various biochemical states is not known, there is qualitative biochemical support for the notion that 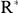-RK affinity decreases systematically with the phosphorylation level of 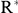 [38]. It is shown in Buczylko et al. [39], that phosphorylated RK has significantly lower ability to phosphorylate already phosphorylated 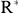 than unphosphorylated 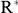. Moreover, Mendez et al. [3] showed that the rate of 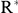 deactivation depends not on the identity of the available sites, but on their total number. We used the biochemically realistic assumption that the rate of phosphorylation is proportional to the number of serines and threonines still available for RK on the rhodopsin molecule. Mechanistically this means that the probability that upon binding to light-activated rhodopsin RK dissociates without adding another phosphate increases with the number of phosphates present, reaching 1 when all six sites are already phosphorylated. This assumption is consistent with in vivo observations by Mendez et al [3] that the removal of even one or two rhodopsin phosphorylation sites slows down photoresponse inactivation. Note that 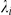 is the rate at which RK phosphorylates 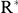 in its 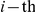 state. It depends on the on-rate of RK binding to 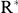 in this state, and the rate of phosphate transfer, which were never separated experimentally and were not separated in our model.

We set the sequence 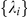 as linearly decreasing by increasing phosphorylation levels, that is the phosphorylation rate is proportional to the total number of the available sites and is independent of their biochemical identity. Thus
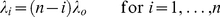
(7)where 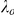 is a rate constant, discussed and calibrated in the section on parameters. Formula Eq:7 can be arrived at by postulating that RK has a fixed affinity for binding to 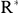 and that each of the phosphorylation sites could be occupied with an equal rate 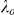. Therefore 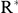 phosphorylation rate depends on the number of phosphorylation sites available for RK. Since 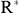 in state 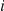 has 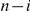 available phosphorylation sites it has a phosphorylation rate 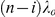. Note that this model, similar to previously proposed ones, is based on the assumption that a single site is phosphorylated as a result of each rhodopsin encounter with RK. This assumption has not been experimentally tested.

### Arr Binding Rate 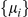 and Affinity of R-Arr

Arrestin binding ensures the timely termination of 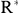 signaling, and it depends on the 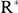-Arr affinity. Several studies [26], [32], [36], suggest that arrestin affinity increases with increasing phosphorylation levels. Note that only the “irreversible” binding that terminates rhodopsin activity is taken into account here; that is the binding which occurs with high enough affinity to make the complex half-life much greater than the time course of the SPR. In a recent study, Vishnivetskiy et al. [20] found that unphosphorylated and mono-phosphorylated 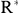 show the same low Arr binding levels. In particular, a single receptor-attached phosphate does not facilitate Arr binding; two are necessary to induce higher affinity interaction, and 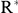 with three phosphates is fully capable of binding Arr with the affinity that makes the interaction essentially irreversible on the time scale of the SPR. Moreover, higher phosphorylation levels do not increase the stability of Arr complex with light-activated rhodopsin [20]. Based on the data in [20], we set the sequence 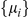 for Arr binding rate by the phosphorylation level as
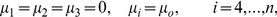
(8)where 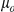 is the Arr binding rate when Arr affinity reaches its maximum after several phosphorylations. Note that 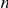 in the model describes arrestin binding that terminates transducin activation. Thus, it reflects the rate of formation of arrestin-rhodopsin complexes that are stable enough to survive significantly longer than the time course of a single photon response analyzed here. Since the stability of arrestin complex with unphosphorylated and mono-phosphorylated rhodopsin is much lower [20], [40]–[42], allowing for arrestin dissociation and consequent rhodopsin reactivation within this time, we set 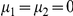. Since 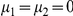, 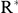 in states 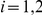 surely transitions to the states 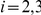 respectively. The data in vivo [3] and in vitro [20] also suggest that two rhodopsin-attached phosphates are not sufficient to induce Arr binding with high enough affinity for rapid deactivation. Therefore, we set 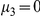.

The effect of the level of rhodopsin phosphorylation on arrestin binding was explored in two studies. Gibson et al [32] concluded that arrestin affinity linearly increases with the level of phosphorylation in the range of 1–4 phosphates per rhodopsin. The authors used preparations of phosphorylated rhodopsin in native disc membranes that are well known to be highly heterogeneous, containing rhodopsin species carrying from zero to seven phosphates (bovine rhodopsin has seven RK phosphorylation sites) [26], [43]. The authors attempted to solve this problem by using several assumptions (that were not experimentally tested) to compute the fraction of rhodopsin molecules with different phosphorylation levels as a function of average phosphorylation, which was the only parameter actually measured [32]. The authors calculations were based on an additional assumption that unphosphorylated rhodopsin does not bind arrestin, even though specific low affinity binding of wild type arrestin to light-activated unphosphorylated rhodopsin in vitro [40], [41], and its role in inactivation of unphosphorylated rhodopsin in vivo [12], [23], [42] was shown. Arrestin binding in this study was measured using “extra Meta II” assay developed by Schleicher et al in 1989 [44]. This assay is based on the stabilization of active Metarhodopsin II state by bound arrestin. The most significant drawback of this assay is that it does not work above 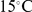. At physiological temperatures extra Meta II is not detectable, even though it is obvious that arrestin effectively quenches rhodopsin signaling in mammals at 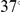
–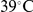. In another study Vishnivetskiy et al [20] separated rhodopsin species with different levels of phosphorylation by chromatofocusing. Importantly, the authors quantitatively determined the presence of particular phospho-rhodopsin species in each fraction by mass-spectrometry of proteolytically removed rhodopsin C-terminus [34], obviating the need for calculations based on untested assumptions. Moreover, the binding assay in this study was performed at physiological temperature, 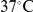. Based on their data, Vishnivetskiy et al concluded that arrestin demonstrates the same low affinity for rhodopsin carrying zero and one phosphate. The presence of two phosphates somewhat increases arrestin affinity, whereas arrestin binds rhodopsin with three, four, five, and six phosphates with the same high affinity, forming physiologically relevant long-lived complexes with stability sufficient for reliable quenching without possibility of reactivation on the time scale of the photoresponse [20]. These conclusions are in remarkable agreement with the work of Mendez et al in genetically modified mice expressing rhodopsin with different number of phosphorylation sites [3]. The authors of this study found that in vivo light activation of rhodopsin carrying zero, one, or two phosphorylation sites yields responses that last for many seconds, whereas rhodopsin carrying three or more phosphorylation sites is inactivated by wild type arrestin with sub-second kinetics [3]. Therefore, we based the modeling on the conclusions of these two studies [3], [20]. The parameters 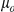 and 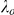 appearing in Eq:7–Eq:8 are calibrated by WT and mutant experimental data, and not by fitting (see section on parameters).

### Random Sojourn Times 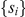 and Random Steps to 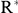 Shutoff

In the state 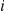, 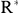 maintains its catalytic activity 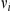 for a random time 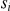, until further phosphorylation or Arr binding. The sojourn times 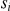, for 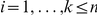 are exponentially distributed random variables with mean 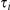. The average lifetime 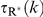 of 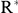 being deactivated after 
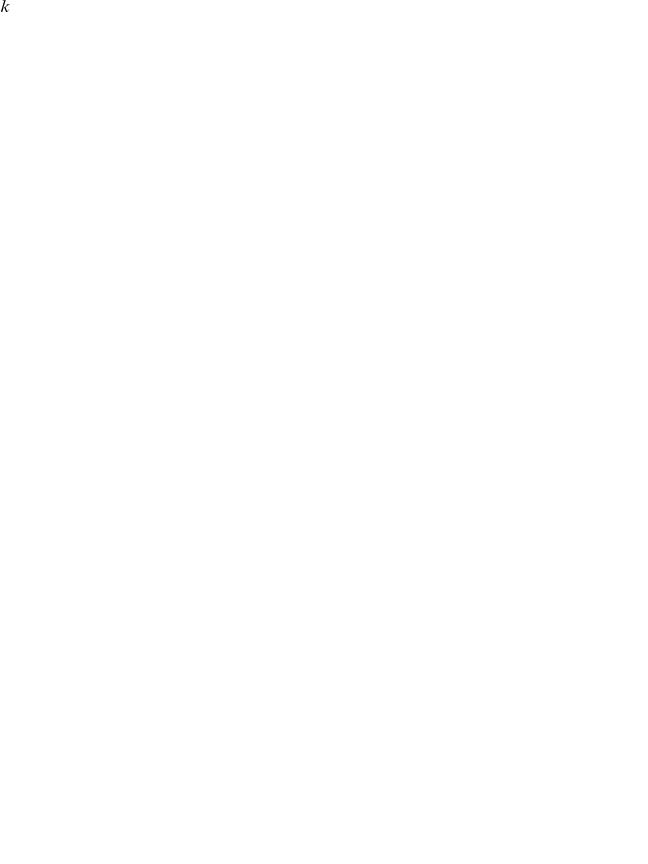
 random biochemical states visited by 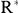 before quenching, and is the sum of the 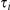 up to 
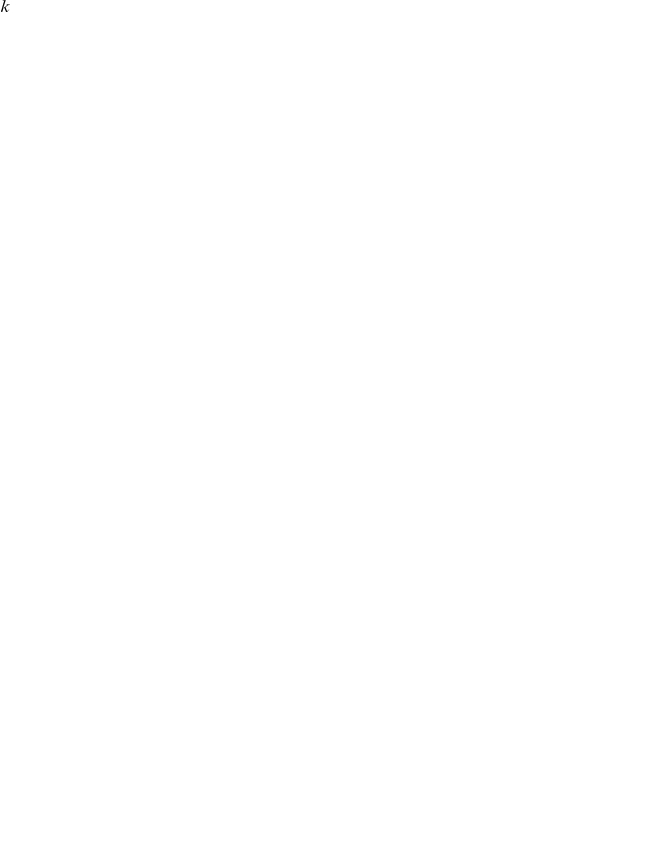
. Thus
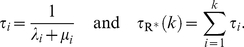
(9)Hence 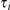 and 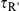 are determined by the biochemistry of the process through the sequences 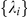 and 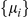.

The number 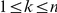 of steps after which 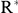 binds to Arr is itself a random variable 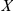. The probability of 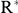 shutoff in 
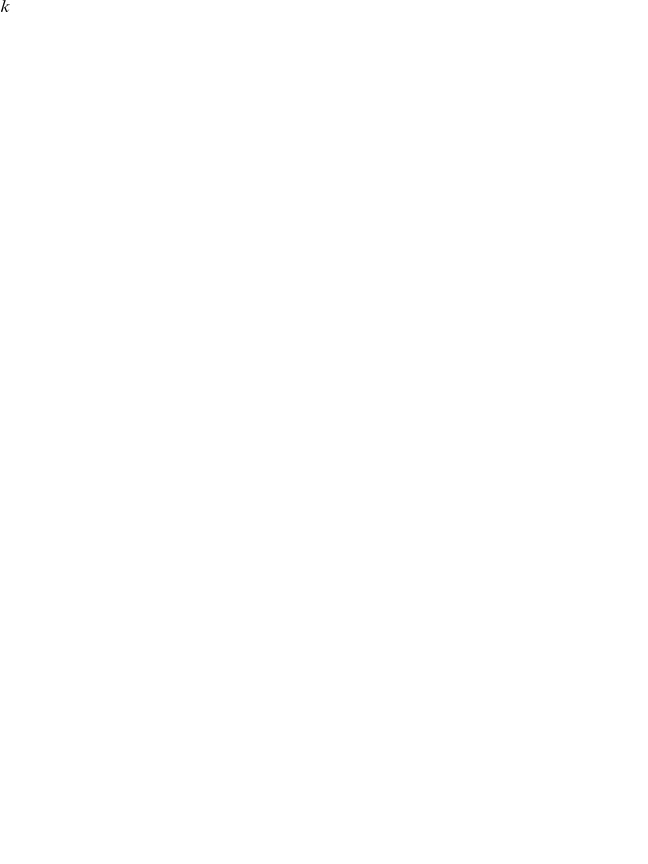
 steps, or equivalently the probability of 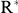 undergoing 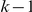 steps of phosphorylation and a final step for Arr binding, is
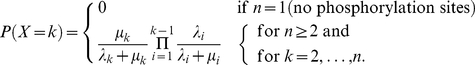
(10)The mean steps of 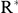 shutoff, or equivalently the expected value of the first moment of 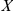, is denoted by 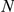 and is given by
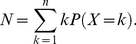
(11)Thus 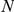 is the mean of the discrete valued random variable 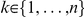.

The lifetime 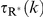 of 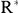 is itself a random variable with expected value
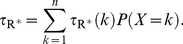
(12)These remarks permit one to detect the pattern of the means 
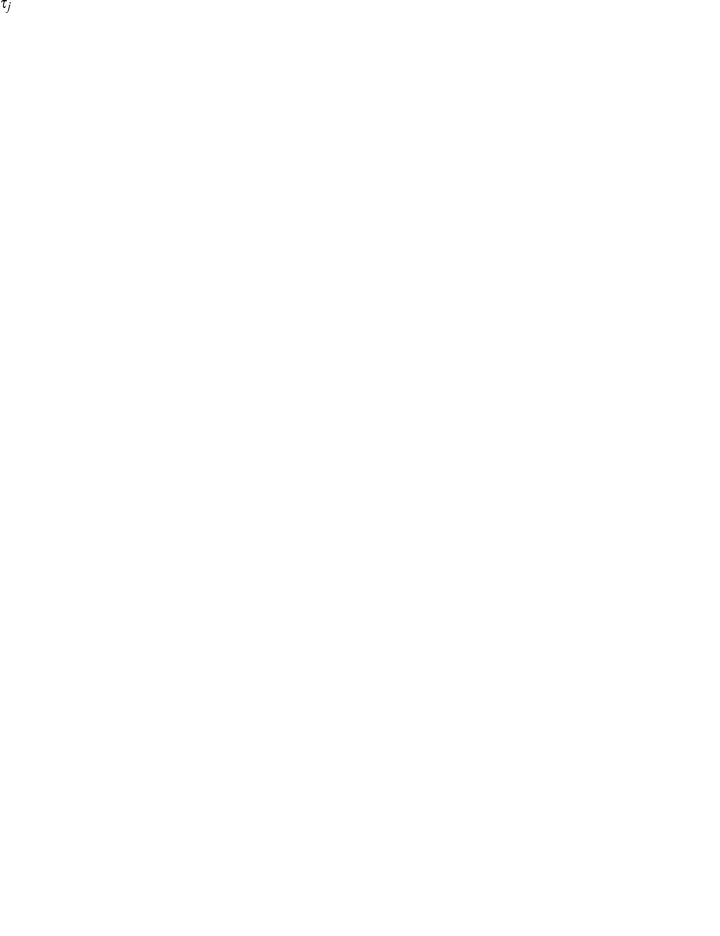
 of the random sojourn times 
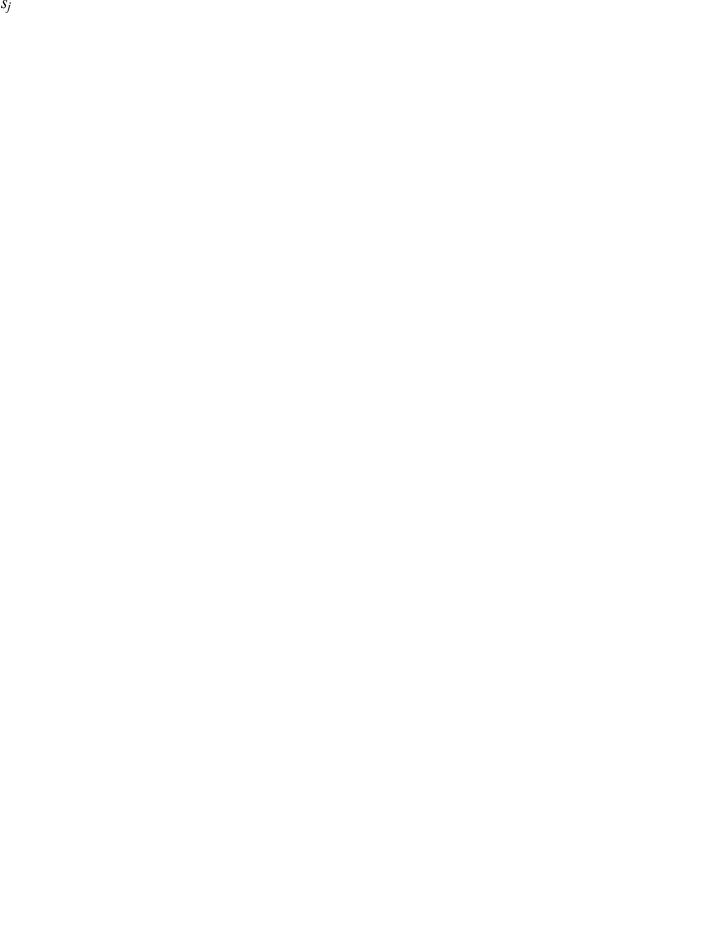
. First, the expected lifetime 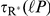 of 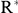, as a function of the number 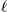 of available phosphorylation sites, decreases with increasing 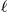; second, the sojourn times 
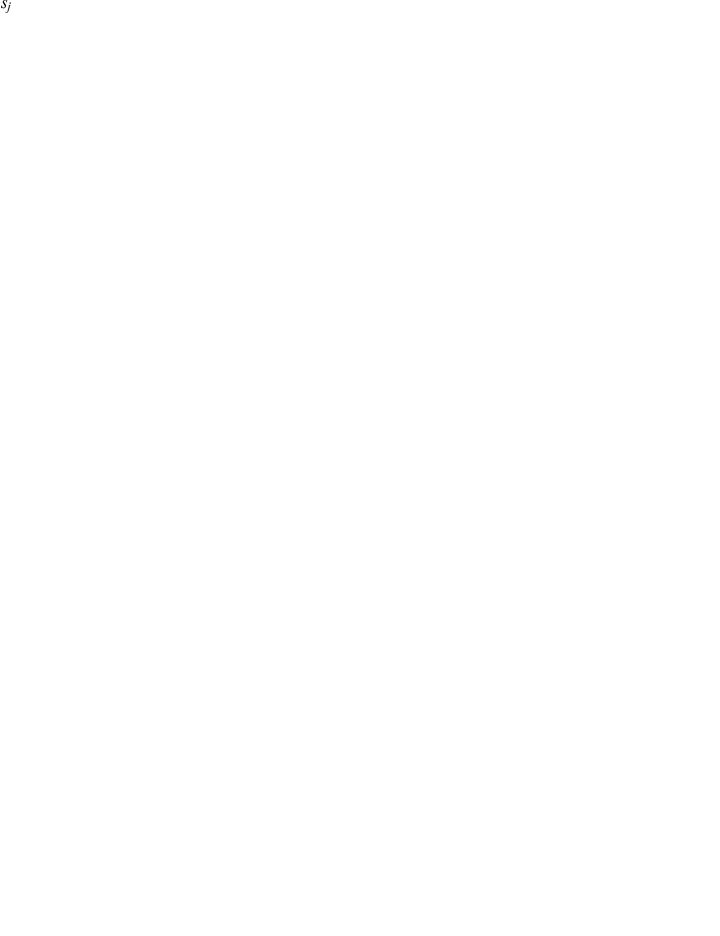
, while increasing in number, each have a smaller mean 
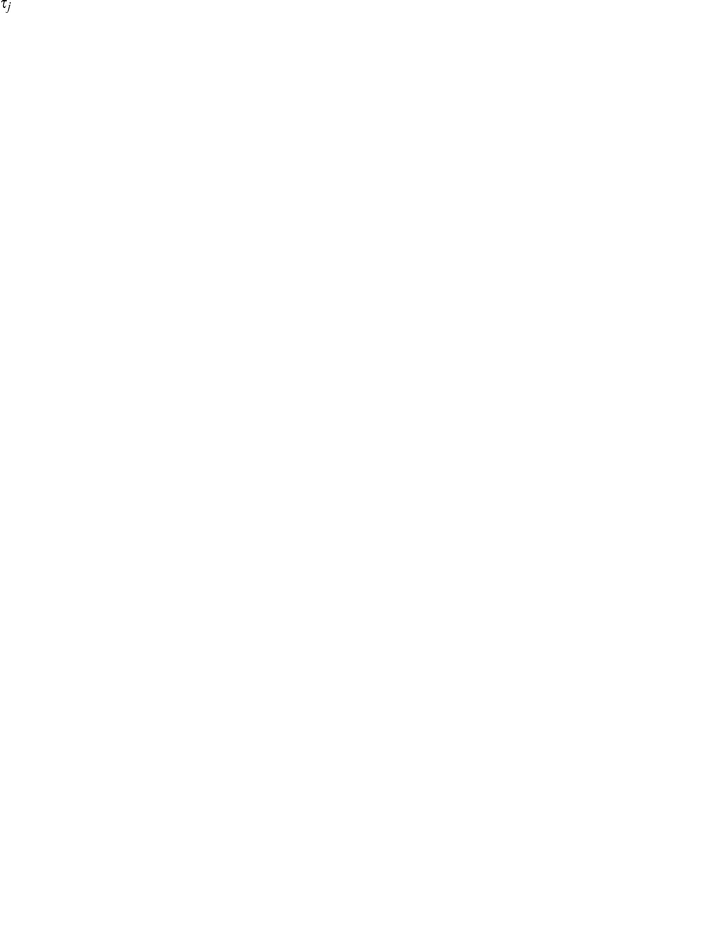
. For example for 3P (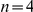), from Eq:7–Eq:9, and Eq:10–Eq:11, one computes
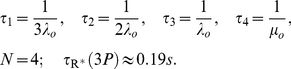
(13)We stress that the number 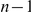 of available phosphorylation sites does not coincide with the mean number 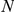 of steps to 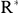 quenching. The number 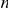 is fixed by the structure of rhodopsin, whereas 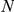 depends on the biochemistry, through the probabilities 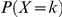.

### Numerical Procedures and Methods

The dynamics of 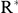 during deactivation is analyzed by the CTMC model in Eq:5, which is numerically integrated in the Matlab platform. Its output is integrated into the spatio-temporal model and its Matlab code introduced in [8], [11]. This produces pointwise values of the effector 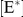, 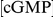 and 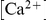 on the ROS and permit one to compute the current response 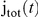 as a function of time and thus the functionals of effector and current in Eq:1–Eq:2.

Simulations were performed for each of the 3 Test Cases. Random numbers are generated according to the exponential distribution of the random sojourn times 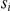, for each of wild type, transgenic and Arr knock-out cases indicated above. The corresponding 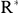 dynamics is computed and the functionals in Eq:1–Eq:2 are evaluated. For each case, after about 5,000 numerical simulations, we compute the mean, the standard deviation and the coefficient of variation (CV) of these functionals.

For WT mice the sequences 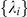 and 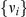 are chosen as in Eq:6–Eq:7. For mice lacking phosphorylation whether by COOH-terminal truncations ([24], [25]) or RK knockout ([1], [3]), in the CTMC one sets 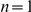 in Eq:5 and 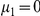, so that 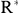 would remain in state 1 with catalytic activity 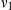 for the whole process. If 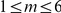 of the six phosphorylation sites are mutated out, we would have a CTMC model with 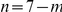. For mice lacking Arr ([24]), we let 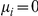, for 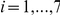 in the CTMC model in Eq:5.

### Parameters

In [37], we have generated a complete, self-consistent set of parameters for the mouse rod phototransduction, calibrated by least square fitting of the model in [8], [9], [11]. to a set of experimental data kindly provided Dr. C. Makino, leading to Table S2 (Text S1). Note that these parameters describe SPR recorded in Locke's solution used in [3], [7], [24], [25], [28], [29], [42], [45]–[49], rather than in Ames' solution used in [1], [2]. For reasons that remain to be elucidated, the latter has greater amplitude and duration, although the recovery in both conditions is rate-limited by transducin inactivation ([28]).

**Figure 4:**
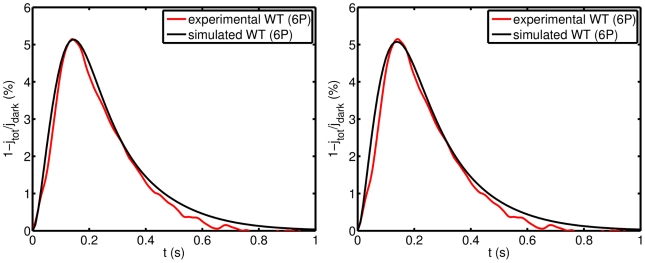
Mouse SPRs by simulation (black) and experiment (red). The simulation is conducted with the parameters shown in Table S2 for the dynamics 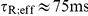 and 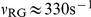 (left), and the dynamics suggested in [28], [30], [31](right). **Left**: Dynamics of 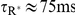 and 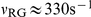. The WT SPR exhibits a maximum of 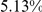, decrease of current at 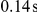 after activation. Experimental data is an average of sets of SPRs kindly provided by C. Makino. **Right**: Same experimental (red) and simulated (black) WT response with 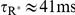 and 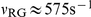. Experimental data kindly provided by C. Makino.

Figure 4 compares the simulated SPR by our model with the parameters of Table S2, and the experimental SPR kindly provided by C. Makino. The new parameters involved in the present investigation are the biochemical sequences 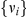, 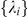 and 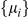. Below we indicate in detail how they have been determined. Their estimated values are reported in Table 4. Given the catalytic rate 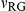 the sequence 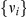 is determined from Eq:6 whence the rate 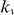 is known.

**Table 4:** CTMC model parameters.

Symbol	Units	Definition	Value		References
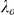	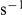	Rhodopsin phosphorylation rate	*10.5*	**19**	computed as in **Determining the Sequences ** 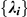 ** and ** 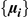
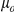	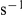	Arresting binding rate	*60*	**120**	computed as in **Determining the Sequences ** 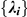 ** and ** 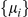
k 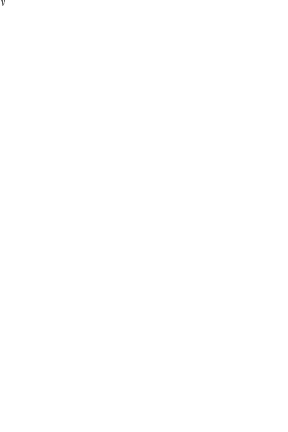	-	Decay constant of catalytic activity of R*	*0.5*	**0.5**	[26]
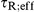	ms	Average lifetime of active R*	*75*	**41**	[*29*], [**28]**, [30], [31****]
N	-	Average number of steps of active R* before shut off	*4.45*	**4.41**	computed from Eq:9–Eq:11

*Italic:* 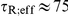 *ms*. **Bold:** 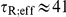 **ms**. The parameters 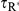 and 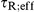 and their equivalence are discussed in 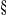 **Parameters**.

#### Estimate of 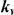


In the experiments of [26] a large pool of G proteins, PDE and cGMP was mixed with a large quantity of rhodopsin 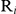 with a known number 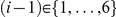 of phosphates. Then the 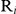 were activated by a brief flash of light to produce a number of isomerizations 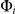 per 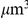, and the rate of depletion cGMP was recorded. Since only three purified proteins are present in this assay, rhodopsin, transducin, and PDE, rapid inactivation mechanisms present in vivo do not operate. Therefore, the number of activated transducin molecules is proportional to the number of light-activated rhodopsins and their activity, and the number of active PDE molecules is proportional to the number of active transducins and does not change in time.

The number of molecules involved, all in the same environment, is so large as to justify a lumped description of the phenomenon by means of standard balance equations
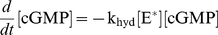
(14)where 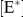 is the number of molecules of fully activated PDE per 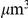, and 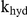, in 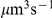 is the rate of hydrolysis of cGMP by 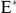. If 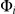 is sufficiently large, the system saturates in the sense that all available molecules of PDE are fully activated. Denoting by 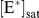 the limiting saturation, one computes
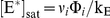
where 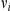 is the activity of 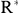 in its 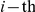 state, and 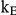 is the decay rate of 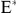. It is assumed that for large 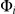 the time to saturation is very small so that, in Eq:14, one approximates 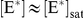. from the second of Eq:14. These remarks in Eq:14 imply
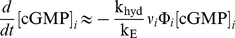
(15)where the indexed 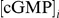 signifies that in principle the solution of such equation depends on the activity of the 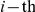 phosphorylation state of 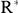. At saturating light levels the rates 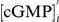 reach a limiting value independent of 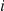. Moreover at such limiting rates the 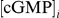 are essentially the same for all 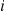, since they are hydrolyzed by 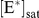. It is reported in [26] that the same rate of depletion 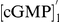 for an experiment with rhodopsin 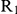 (no phosphates) with activity 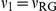, and number of isomerizations 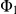, can be obtained from an experiment with rhodopsin 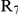 (6 phosphates), and catalytic activity 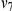, provided the number of isomerizations 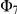 is 10 times larger than 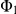. Equation Eq:15 with these data and indicated assumptions yields
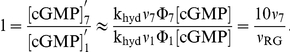
From this and Eq:6 one computes 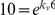 or 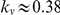.

#### Determining the sequences 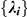 and {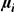}

The modeling assumptions contained in Eq:7–Eq:8 reduce these sequences to the determination of 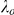 and 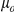. These are constrained by Eq:12. Therefore, if the expected lifetime 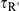 of activated rhodopsin, given by Eq:12, is experimentally estimated, then only one of these parameters, 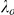 or 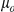, is independent. When 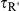 is fixed at a particular value, the remaining free parameter is estimated against the experimental results of [24] for SPR in transgenic mice lacking arrestin, as follows. In the absence of arrestin, the activated rhodopsin gets phosphorylated from one level to another until all its six sites are occupied. Its activity is reduced with phosphorylation, and kept fixed after the last phosphorylation, for the remainder of the process. Such activity causes the response tail to maintain almost a half of its peak value (Figure 2C,D). Thus 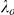 and 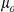 are constrained by Eq:8 and by the requirement that putting 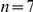 in the CTMC Eq:5 and 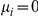 for all 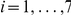 the time asymptotic current suppression is about 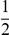 of the peak current suppression.

It is worth stressing that the parameter calibration has been effected by enforcing at the same time, the WT response, the response of transgenic mice lacking arrestin, and by linking the rates 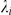 and 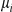 to the experimental value of 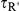 by Eq:12. It is also worth noting that here we simulated the first 3 s of the photoresponse. Light-activated rhodopsin can also be inactivated in an RK- and Arr-independent manner via thermal decay to opsin with very low activity towards transducin. Since this process is very slow, with half-life of 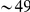 s in mouse photoreceptors [13], we did not take it into account in our modeling.

#### On the parameters 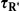 and 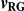


In Table S2, 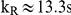 is the reciprocal of the experimental value of the average lifetime of 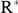 for WT mouse, denoted by 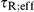, and determined as the time constant of an exponential decay function used to approximate 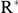 lifetime ([28], [30], [31]). As such 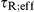 is an “effective lifetime”. In [29] 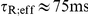 is an upper limit of 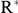 lifetime.

The expected value 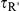 of 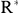 lifetime given by Eq:12 is the average of the times it takes 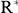 to be quenched after 
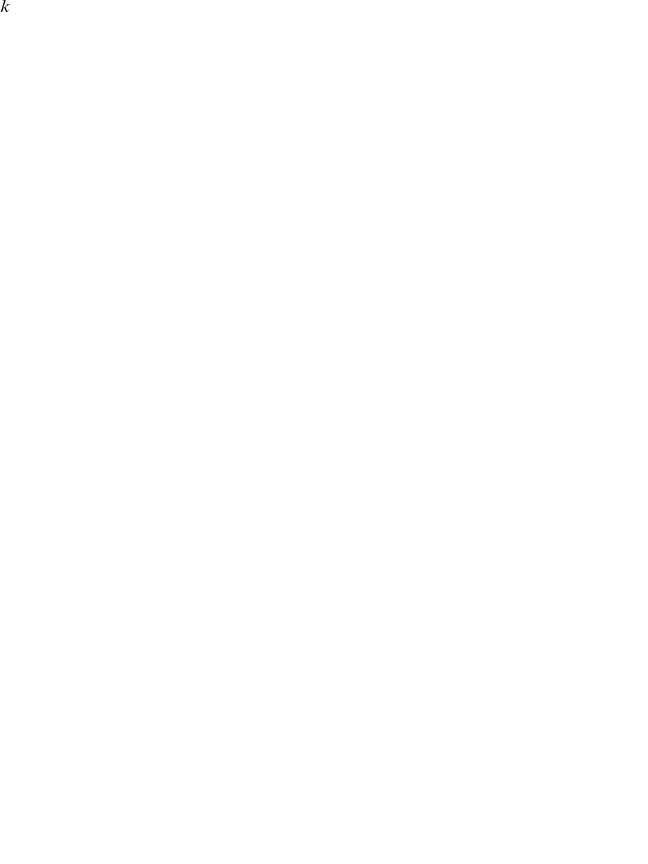
 steps, weighted by the probabilities of quenching after 
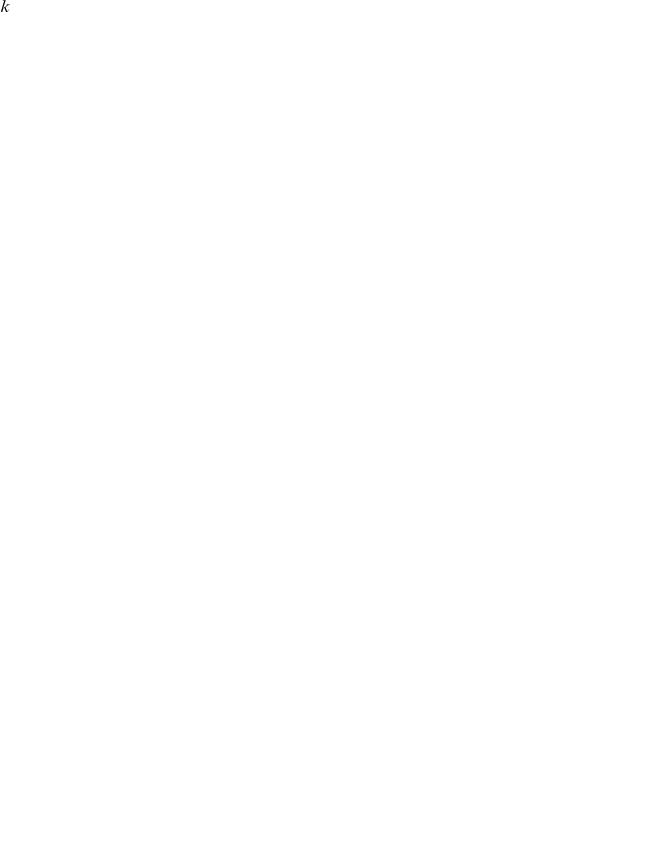
 steps. This number depends only on the biochemistry defined by the sequences 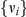 and 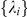.

If one knew these sequences a-priori, no knowledge of 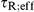 would be needed. The numerical value of the latter is used to generate an extra link between the parameters 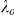 and 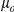, for WT mouse (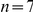) to reduce the number of free parameters. Thus the underlying assumption is that for WT mouse, the expected value 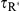 of the random variable 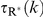 is *numerically* comparable with the experimentally measured numerical value of 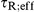, and thus one sets *numerically* 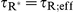. For this reason, when referring to WT mouse, and only in this case, we use 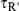 and 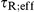 interchangeably. However for genetically modified mice (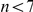) the expected values of 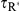 given by formula Eq:13 differ from 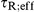.

It should be pointed out however that 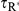 is a derived parameter through formula Eq:12. It is the biochemistry that determines 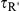 through the RK on-rates 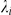 and the Arr on-rates 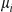. Thus 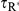 changes in genetically modified mice according to the number of mutated sites, and the resulting biochemistry.

Several recent studies give a lower estimate 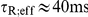 for WT [30]. Because of the importance of this parameter we have reproduced our simulations also for 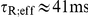, and found no appreciable difference in the numerical values or the general pattern of the resulting variability functionals (see Figure S1 and Tables S1,S3 of the supplementary material). Thus, the functional conclusions of this study do not depend on the numerical value of 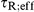.

By taking a shorter 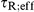, the SPR for WT mouse can be reproduced by imposing a larger catalytic rate 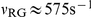 (i.e., activation of transducin by rhodopsin every 2 ms). A few other parameters have been slightly modified the most noticeable of which are the RK on-rate 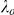 and the Arr on-rate 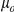. Using the pair 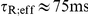 and 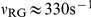 and computing 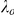 and 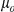 as indicated in the previous section, one estimates 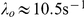 and 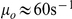. Using the pair 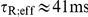 and 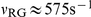, one estimates 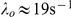 and 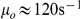. All the indicated simulations have been run for both sets of parameters with no appreciable difference in the results (Figure 1 and Tables 2–3, and Figure S1 and Table S1,S3 in the supplementary material).

The value of 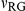 reported in Table S2 corresponds to the value 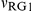 as calculated from Eq:6 for 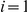. The (random) production of 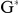 by 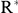 in its 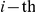 state is 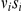. If shutoff occurs in 
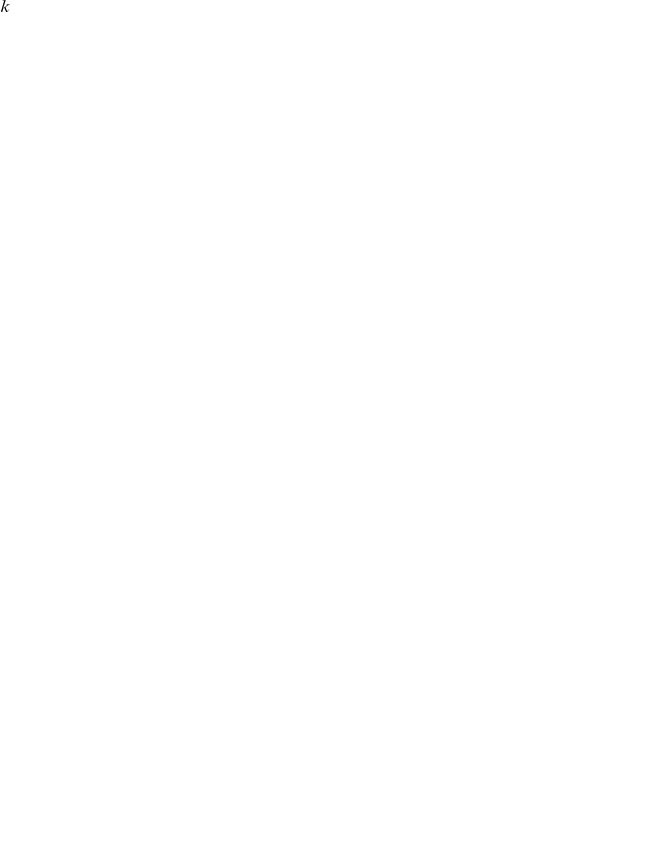
 steps, the average activity over these steps is
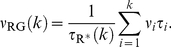
(16)This is a random variable whose expectation is the expected (average) activity 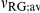 of the process
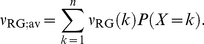
(17)A calculation for 6P and 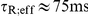 gives 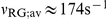. This value is within the published range of average 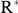 activity as discussed in [37]. A similar calculation for 6P and the faster dynamics 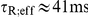 gives 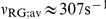.

Remaining in the context of WT mouse, the shortening of 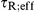 from 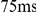 to 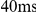 forces a faster dynamics so that the total activity remains unchanged. One verifies that the activity 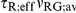 remains the same for both values of 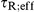. The two dynamics generate two different biochemical sequences, say for example
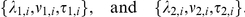
An examination of Table 3 and Table S3 in the supplementary material reveals that, for each fixed 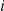 the products 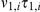 and 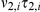, are essentially the same for the two dynamics. Thus the total 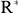 activity is redistributed in “equal bits”, although in different time intervals 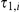 and 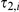, and different catalytic activities 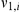 and 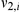. The theoretical formula Eq:3 then gives
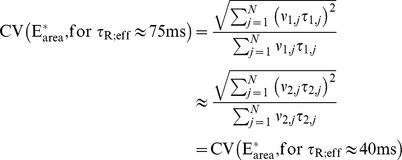



This explains why the CVs of 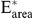, and hence those of 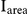 are so similar, for each of these dynamics. A further examination of Table 3 and Table S3 of the supplementary material shows that to the total activity 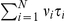 contribute essentially only the first few steps, the remaining ones being negligible. In view of the theoretical formula Eq:3, this is further evidence that increasing the number of steps, does not significantly decrease the CV(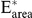).

The main difference between the CVs in Table 1 and Table S1 in the supplementary material occurs for the pointwise functionals 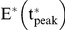 and 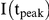, which depend on a point evaluation at 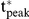 and 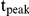 respectively, and do not depend on the total, integral activity up to time 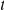.

#### The average number of steps to shutoff

This number is computed from Eq:10–Eq:11, and therefore it is not expected to be an integer.

For 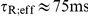 and 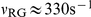 and the corresponding 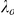 and 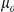, one estimates 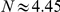. For the faster dynamics 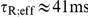, and 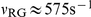, one estimates 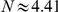.

The parameters of Table S2 and Table 4 have been slightly calibrated to satisfy simultaneously all the indicated constraints. Figure 4 compares the simulated and experimental single photon response in WT mouse. The simulations for transgenic mouse in Figure 2 are compared with the experimental data of [1], [3], [23]–[25]. The dynamics of the WT mouse SPR reported in [1], is, in absolute time, slower than that reported in [3], [23]–[25]. The difference might be the result of using different solutions for single cell recording [2], [28]. The underlying mechanism of this phenomenon remains unknown. To achieve a functional comparison with all these contributions we have reported our simulations in units of 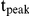 and likewise we have rescaled the graphs reported in [3], [23] in units of their own 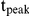. The output (given in pA in the original papers) has been rescaled in relative current suppression 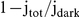.

## Supporting Information

Figure S1 Comparing the CVs of the total activated effectors at time t with the CVs of the total relative charge up to time t.
(0.05 MB PDF)

Figure S2 Simulations SPR for mutant phosphorylation sites of R* or with Arr knockout.
(0.08 MB PDF)

Table S1 CVs of effector and current for WT and mutant mouse SPR.
(0.04 MB PDF)

Table S2 Parameter table for WT SPR.
(0.06 MB PDF)

Table S3 Table of distribution of activities for WT and mutant mouse SPR.
(0.03 MB PDF)

Text S1 References.
(0.04 MB PDF)
