# Supplementary material for: Kinetics of Rhodopsin Deactivation and Its Role in Regulating Recovery and Reproducibility of Rod Photoresponse
Source: PLoS Comput Biol. 2010 Dec 16;6(12):e1001031. doi: 10.1371/journal.pcbi.1001031 (PMC3002991; doi:10.1371/journal.pcbi.1001031)
Supplement: Figure S1 — Comparing the CVs of the total activated effectors at time t with the CVs of the total relative charge up to time t. (0.05 MB PDF) [file pcbi.1001031.s001.pdf]

CV of $\mathrm{E}^*_{\mathrm{int}}(t)$ and $\mathrm{I}_{\mathrm{int}}(t)$ for WT and Transgenic Mice for $\tau_{\mathrm{R;eff}} = 40$ ms and $\nu_{\mathrm{RG}} = 575\mathrm{s}^{-1}$

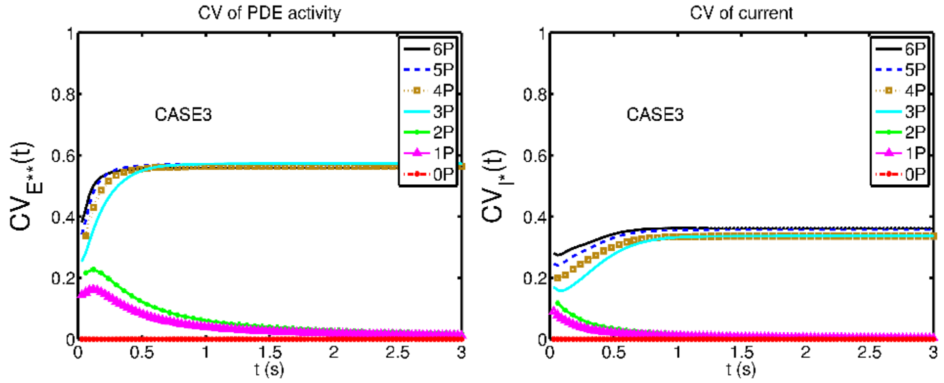

Figure S1. Comparing the CVs of the total activated effectors $\mathrm{E}^*_{\mathrm{int}}(t) = \int_0^t E^*(s)ds$ at time t with the CVs of the total relative charge $\mathrm{I}_{\mathrm{int}}(t) = \int_0^t I(s)ds$ up to time t. All simulations assume both the sojourn time and the number of R^* shutoff steps as random (Case 3 of Test Cases). The CVs of both $\mathrm{E}^*_{\mathrm{int}}(t)$ and $\mathrm{I}_{\mathrm{int}}(t)$ stabilize asymptotically for three or more phosphorylation sites (3P–6P). A CV of about 60% for $\mathrm{E}^*_{\mathrm{int}}(t)$ at times past the peak time is reduced to a CV of about 40% for the corresponding photocurrent $\mathrm{I}_{\mathrm{int}}(t)$. This points to an intrinsic variability reduction effect of the diffusion part of the process. The simulation is conducted with the parameters shown in Table S2 with $\tau_{\mathrm{R}^*} \approx 40$ms and $\nu_{\mathrm{RG}} \approx 575\mathrm{s}^{-1}$.
