# Supplementary material for: Kinetics of Rhodopsin Deactivation and Its Role in Regulating Recovery and Reproducibility of Rod Photoresponse
Source: PLoS Comput Biol. 2010 Dec 16;6(12):e1001031. doi: 10.1371/journal.pcbi.1001031 (PMC3002991; doi:10.1371/journal.pcbi.1001031)
Supplement: Figure S2 — Simulations SPR for mutant phosphorylation sites of R* or with Arr knockout. (0.08 MB PDF) [file pcbi.1001031.s002.pdf]

SPR for Transgenic Mouse for $\tau_{\mathrm{R;eff}} = 40$ ms and $\nu_{\mathrm{RG}} = 575\mathrm{s}^{-1}$

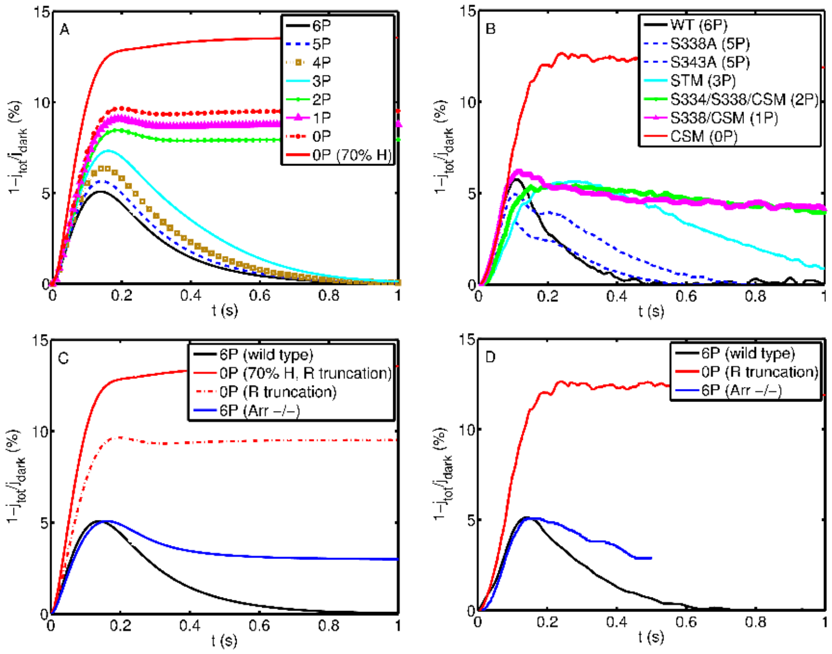

Figure S2. Simulations SPR for mutant phosphorylation sites of R^***, or with Arr knockout. Panel A**: Simulated SPRs for rhodopsin with a number $n = 0, 1, ..., 6$ of available phosphorylation sites (thus $(6 - n)$ sites are mutant); **Panel B**: Reproduction of data from [36] showing SPRs from mutant mice with different phosphorylation sites. CSM: completely substituted mutant (0P); STM: serine triple mutant (3P); S338A: mutant lacking S338 (5P); S343A: mutant lacking residue S343 (5P); S338/CSM: one site (S338) was restored in the CSM (1P); S334/S338/CSM: two sites (S334 and S338) were restored in the CSM (2P); Mutant rhodopsins bearing zero, one (S338), or two (S334/S338) phosphorylation sites generated single-photon responses with greatly prolonged durations. Responses from rods expressing mutant rhodopsins bearing more than two phosphorylation sites declined along smooth, reproducible time courses; the rate of recovery increased with increasing numbers of phosphorylation sites; **Panel C**: Simulated SPRs with no phosphorylation site (0P), lacking arrestin (−/−), and wild type (WT); **Panel D**: Reproduction of the SPRs from rod with C terminal truncation, lacking arrestin (−/−), and wild type (+/+) [41]. With arrestin absent, the flash response displayed a rapid partial recovery followed by a prolonged final phase. This behavior indicates that an arrestin-independent mechanism initiates the quench of rhodopsin's catalytic activity and that arrestin completes the quench.
