# Supplementary material for: Kinetics of Rhodopsin Deactivation and Its Role in Regulating Recovery and Reproducibility of Rod Photoresponse
Source: PLoS Comput Biol. 2010 Dec 16;6(12):e1001031. doi: 10.1371/journal.pcbi.1001031 (PMC3002991; doi:10.1371/journal.pcbi.1001031)
Supplement: Table S1 — CVs of effector and current for WT and mutant mouse SPR. (0.04 MB PDF) [file pcbi.1001031.s003.pdf]

CVs of E^*_{area} and I_{area} of the WT and Mutant SPR for the Dynamics $\tau_{R;eff} \approx$ 40 ms and $\nu_{RG} \approx 575s^{-1}$

Table S1. Coefficients of variation, $\tau_{R^*} \approx 40$ ms

Sites		0P	1P	2P	3P	4P	5P	6P(WT)
$E^*(t^*_{peak})$	**Case1**	0.00	0.05	0.13	0.40	0.43	0.4 7	0.51
	**Case2**	0.00	0.00	0.00	0.00	0.00	0.00	0.00
	**Case3**	0.00	0.05	0.11	0.40	0.45	0.48	0.49
E^*_{area}	**Case1**	0.00	0.01	0.02	0.57	0.56	0.56	0.58
	**Case2**	0.00	0.00	0.00	0.00	0.01	0.02	0.02
	**Case3**	0.00	0.01	0.02	0.57	0.56	0.57	0.56
$^{(\mathbf{teor})}E^*_{area}$	**Case1**	-	-	-	0.56	0.54	0.53	0.52
$I(t_{peak})$	**Case1**	0.00	0.03	0.05	0.19	0.25	0.2 9	0.32
	**Case2**	0.00	0.00	0.00	0.00	0.00	0.01	0.01
	**Case3**	0.00	0.03	0.05	0.20	0.25	0.29	0.31
I_{area}	**Case1**	0.00	0.00	0.01	0.33	0.34	0.35	0.37
	**Case2**	0.00	0.00	0.00	0.00	0.01	0.01	0.02
	**Case3**	0.00	0.00	0.01	0.34	0.34	0.36	0.36

CV (σ/μ) calculated for a 3 s simulation and 5000 trials for each of **Case 1**: Fixed number of steps to R^* shutoff and random sojourn times s_i; **Case 2**: Fixed sojourn times s_i and random number of steps to R^*; **Case 3**: Both sojourn times s_i and R^* shutoff steps are random. The parameters τ_{R^*} and $\tau_{R;eff}$ and their equivalence for WT mouse are discussed in the section § **Parameters**. The theoretical values of $^{(teor)}E^*_{area}$ are reported for 3-6P as the theoretical formula of Eq:3-4 is valid only for these cases.
