# Supplementary material for: Kinetics of Rhodopsin Deactivation and Its Role in Regulating Recovery and Reproducibility of Rod Photoresponse
Source: PLoS Comput Biol. 2010 Dec 16;6(12):e1001031. doi: 10.1371/journal.pcbi.1001031 (PMC3002991; doi:10.1371/journal.pcbi.1001031)
Supplement: Table S2 — Parameter table for WT SPR. (0.06 MB PDF) [file pcbi.1001031.s004.pdf]

A Parameter Table for the WT SPR for the Dynamics $\tau_{\mathrm{R;eff}} \approx 75$ ms and $\nu_{\mathrm{RG}} \approx 330\mathrm{s}^{-1}$ and the Faster Dynamics $\tau_{\mathrm{R;eff}} \approx 40$ ms and $\nu_{\mathrm{RG}} \approx 575\mathrm{s}^{-1}$

Table S2 collects the simulation parameters for the two indicated dynamics. The ones in black, equal to those of Table S2 reflect the slower dynamics $\tau_{\mathrm{R;eff}} \approx 75$ ms and $\nu_{\mathrm{RG}} \approx 330\mathrm{s}^{-1}$ and those in **bold** are those that need to be modified to accomodate the faster dynamics $\tau_{\mathrm{R;eff}} \approx 40$ ms and $\nu_{\mathrm{RG}} \approx 575\mathrm{s}^{-1}$.

Table S2. Model parameters in mouse phototransduction.
BLACK: $\tau_{\mathrm{R}^*} = 75$ ms **Bold:** $\tau_{\mathrm{R}^*} = 41$ **ms**

Symbol	Units	Definition	Value	References
$\alpha_{\max}$	$\mu\mathrm{Ms}^{-1}$	Maximum rate of cGMP synthesis at low Ca^{2+} concentration	76.5	[1,2]
$\alpha_{\max}/\alpha_{\min}$	-	Suppression ratio of α from high to low Ca^{2+} concentration	13.9	[1–3]
A_{inc}	$\mu\mathrm{m}^2$	Area of the incisure	0.0403	
β_{dark}	s^{-1}	Rate of cGMP hydrolysis by dark activated PDE	2.9	[2,4,5]
B_{cG}	-	Buffering power of cytoplasm for cGMP	1	[6–8]
B_{Ca}	-	Buffering power of cytoplasm for Ca^{2+}	20	[6,9,10]
c_{GE}	-	Coupling coefficient from G^* to E^*	1	[8,11]
$[\mathrm{cGMP}]_{\mathrm{dark}}$	μM	Concentration of cGMP in the dark	3.80 (**3.37**)	[2,3,6–8,12–14]
$[\mathrm{Ca}^{2+}]_{\mathrm{dark}}$	nM	Concentration of Ca^{2+} in the dark	344 (**394**)	[4,8,15–22]
D_{cG}	$\mu\mathrm{m}^2\mathrm{s}^{-1}$	Diffusion coefficient for cGMP	120	[23–25]
D_{Ca}	$\mu\mathrm{m}^2\,\mathrm{s}^{-1}$	Diffusion coefficient for Ca^{2+}	15	[9]
D_{E^*}	$\mu\mathrm{m}^2\,\mathrm{s}^{-1}$	Diffusion coefficient for activated PDE	1.2	[7]
D_{G^*}	$\mu\mathrm{m}^2\,\mathrm{s}^{-1}$	Diffusion coefficient for activated G protein	2.2	[7]
D_{R^*}	$\mu\mathrm{m}^2\,\mathrm{s}^{-1}$	Diffusion coefficient for activated Rh	1.5	[7]
ε	nm	Disk thickness	14.5	[7,26,27]
η	nm	Volume-to-surface ratio	7.25	
$\mathcal{F}$	$\mathcal{C}\,\mathrm{mol}^{-1}$	Faraday's constant	96500	
f_{Ca}	-	Fraction of cGMP-activated current carried by Ca^{2+}	0.06	[1,5,8,28,29]
H	μm	Height of ROS	23.6	[8,26,30,31]
j_{dark}	pA	Dark current	10.9 (**12.21**)	[1,3,4,8,32–41]
$\mathrm{j}_{\mathrm{cG}}^{\max}$	pA	Maximum cGMP-gated channel current	3550	
$\mathrm{j}_{\mathrm{ex}}^{\mathrm{sat}}$	pA	Saturated exchanger current	1.8	[42–44]
k_{cat}/K_m	$\mu\mathrm{M}^{-1}\mathrm{s}^{-1}$	Hydrolytic efficiency of activated PDE dimer	540	[7,11,45]
$k_{\sigma;hyd}$	$\mu\mathrm{m}^3\mathrm{s}^{-1}$	Surface hydrolysis rate of cGMP by dark-activated PDE	2.8×10^{-5}	
$k^*_{\sigma;hyd}$	$\mu\mathrm{m}^3\mathrm{s}^{-1}$	Surface hydrolysis rate of cGMP by light-activated PDE	0.9	

Continued on next page

Table S2 – continued from previous page

Symbol	Units	Definition	Value	References
k_E	s^{-1}	Rate constant for inactivation of PDE	6.5	[4, 5, 46, 47]
k_R	s^{-1}	Rate constant for inactivation of Rh	13.3 **(24.39)**	[4, 10, 47]
k_{T^*E}	$\mu m^2 s^{-1}$	Kinetic constant describing the formation of $T^* - E$ complex and thus the production of E^*	1	[48]
K_{cyc}	nM	Half-saturating $[Ca^{2+}]$ for GC activity	100	[1, 2, 4, 5]
K_{cG}	μM	[cGMP] for half maximum cGMP-gated channel opening	20	[8]
K_{ex}	μM	$[Ca^{2+}]$ for half maximum exchanger channel opening	1.6	[8, 43]
l_b	μm	Width of the incisure	0.2593	[49]
l_r	μm	Length of the incisure	0.3111	[49]
ν	–	Ratio between interdiscal space and disk thickness	1	[7, 8, 11, 26]
$\nu\varepsilon$	nm	Interdiscal space	14.5	[7, 26, 30, 31]
ν_{RG}	s^{-1}	Rate of transducin formation per fully activated Rh	330 **(575)**	[6, 8, 11, 50]
n	#	Number of disks	814	
n_{inc}	#	Number of incisures	1	[7, 26, 49]
N_{Av}	$\# mol^{-1}$	Avogadro number	6.02×10^{23}	
m_{cyc}	-	Hill coefficient for GC effect	2	[1–5]
m_{cG}	-	Hill coefficient for cGMP-gated channel	3.5 **(3.2)**	[2–4, 7, 38]
$[PDE]_\sigma$	$\# \mu m^{-2}$	Surface density of dark-activated PDE	750	[7, 8, 27, 30, 51]
R	μm	Radius of disk	0.7	[7, 8, 26, 27, 30, 31, 52, 53]
σ	-	Ratio between outer shell thickness and disk thickness	15/14.5	
$\sigma\varepsilon$	nm	Distance between the disk rim and the plasma membrane (outer shell thickness)	15	[7, 24]
Σ_{rod}	μm^2	Lateral surface area of ROS	103.8	
V_{cyt}	μm^3	Cytoplasmic volume	18.16	
