# Supplementary material for: Kinetics of Rhodopsin Deactivation and Its Role in Regulating Recovery and Reproducibility of Rod Photoresponse
Source: PLoS Comput Biol. 2010 Dec 16;6(12):e1001031. doi: 10.1371/journal.pcbi.1001031 (PMC3002991; doi:10.1371/journal.pcbi.1001031)
Supplement: Table S3 — Table of distribution of activities for WT and mutant mouse SPR. (0.03 MB PDF) [file pcbi.1001031.s005.pdf]

Table S3. The sequences $\nu_i \tau_i$ for the dynamics of $\tau_{\mathrm{R;eff}} \approx 40$ ms and $\nu_{\mathrm{RG}} \approx 575 \mathrm{s}^{-1}$

**6P (WT)**	N	4.41						
	$\tau_{\mathbf{i}}$	8.77	10.53	13.16	5.65	6.33	7.19	8.33
	$\nu_{\mathbf{i}}$	575.00	348.76	211.53	128.30	77.82	47.20	28.63
	$\tau_{\mathbf{i}}\nu_{\mathbf{i}}$	5.04	3.67	2.78	0.72	0.49	0.34	0.24
**5P**	N	4.27						
	$\tau_{\mathbf{i}}$	10.53	13.16	17.54	6.33	7.19	8.33	
	$\nu_{\mathbf{i}}$	575.00	348.76	211.53	128.30	77.82	47.20	
	$\tau_{\mathbf{i}}\nu_{\mathbf{i}}$	6.05	4.60	3.70	0.81	0.56	0.39	
**4P**	N	4.14						
	$\tau_{\mathbf{i}}$	13.16	17.54	26.32	7.19	8.33		
	$\nu_{\mathbf{i}}$	575.00	348.76	211.53	128.30	77.82		
	$\tau_{\mathbf{i}}\nu_{\mathbf{i}}$	7.57	6.12	5.57	0.92	0.65		
**3P**	N	4						
	$\tau_{\mathbf{i}}$	17.54	26.32	52.63	8.33			
	$\nu_{\mathbf{i}}$	575.00	348.76	211.53	128.30			
	$\tau_{\mathbf{i}}\nu_{\mathbf{i}}$	10.08	9.18	11.13	1.07			
**2P**	N	3						
	$\tau_{\mathbf{i}}$	26.32	52.63	∞				
	$\nu_{\mathbf{i}}$	575.00	348.76	211.53				
	$\tau_{\mathbf{i}}\nu_{\mathbf{i}}$	15.13	18.36	∞				
**1P**	N	2						
	$\tau_{\mathbf{i}}$	52.63	∞					
	$\nu_{\mathbf{i}}$	575.00	348.76					
	$\tau_{\mathbf{i}}\nu_{\mathbf{i}}$	30.26	∞					
**0P**	N	1						
	$\tau_{\mathbf{i}}$	∞						
	$\nu_{\mathbf{i}}$	575.00						
	$\tau_{\mathbf{i}}\nu_{\mathbf{i}}$	∞						

The sequences ν_i (s^{-1}), τ_i (ms) and the average number N of steps to shutoff of R^*, for WT and mutant mice, computed from Eq:9–Eq:11. Computation for the dynamics of $\tau_{\mathrm{R;eff}} \approx 40$ ms and $\nu_{\mathrm{RG}} \approx 575\mathrm{s}^{-1}$. The parameters τ_{R^*} and $\tau_{\mathrm{R;eff}}$ and their equivalence are discussed in § **Parameters**.
