# Supplementary material for: Kinetics of Rhodopsin Deactivation and Its Role in Regulating Recovery and Reproducibility of Rod Photoresponse
Source: PLoS Comput Biol. 2010 Dec 16;6(12):e1001031. doi: 10.1371/journal.pcbi.1001031 (PMC3002991; doi:10.1371/journal.pcbi.1001031)
Supplement: Text S1 — References. (0.04 MB PDF) [file pcbi.1001031.s006.pdf]

References

1. Calvert PD, Govardovskii VI, Krasnoperova N, Anderson RE, Lem J, et al. (2001) Membrane protein diffusion sets the speed of rod phototransduction. Nature 411: 90-94.

2. Tsang SH, Burns ME, Calvert PD, Gouras P, Baylor DA, et al. (1998) Role for the target enzyme in deactivation of photoreceptor G protein in vivo. Science 282: 117-121.

3. Burns ME, Mendez A, Chen J, Baylor DA (2002) Dynamics of cyclic GMP synthesis in retinal rods. Neuron 36: 81-91.

4. Chen CK, Burns ME, He W, Wensel TG, Baylor DA, et al. (2000) Slowed recovery of rod photoresponse in mice lacking the GTPase accelerating protein RGS9-1. Nature 403: 557-560.

5. Makino CL, Dodd RL, Chen J, Burns ME, Roca A, et al. (2004) Recoverin regulates light-dependent phosphodiesterase activity in retinal rods. J Gen Physiol 123: 729-741.

6. Nikonov S, Lamb TD, Pugh ENJ (2000) The role of steady phosphodiesterase activity in the kinetics and sensitivity of the light-adapted salamander rod photoresponse. J Gen Physiol 116: 795-824.

7. Pugh ENJ, Lamb T (1993) Amplification and kinetics of the activation steps in phototransduction. Biochim Biophys Acta 1141: 111-149.

8. Pugh ENJ, Lamb TD (2000) Phototransduction in vertebrate rods and cones: molecular mechanisms of amplification, recovery and light adaptation, St. Louis: Elsevier Science, volume 3 of *Handbook of Biological Physics*, chapter 5. pp. 183-255.

9. Nakatani K, Chen C, Koutalos Y (2002) Calcium diffusion coefficient in rod photoreceptor outer segments. Biophys J 82: 728-739.

10. Nikonov S, Engheta N, Pugh ENJ (1998) Kinetics of recovery of the dark-adapted salamander rod photoresponse. J Gen Physiol 111: 7-37.

11. Leskov IB, Klenchin VA, Handy JW, Whitlock GG, Govardovskii VI, et al. (2000) The gain of rod phototransduction: reconciliation of biochemical and electrophysiological measurements. Neuron 27: 525-537.

12. Lamb TD, Pugh ENJ (1992) A quantitative account of the activation steps involved in phototransduction in amphibian photoreceptors. J Physiol 449: 719-758.

13. Pugh ENJ, Duda T, Sitaramayya A, Sharma RK (1997) Photoreceptor guanylate cyclases: a review. Biosci Rep 17: 429-473.

14. Pugh ENJ, Lamb T (1990) Cyclic GMP and calcium: the internal messengers of excitation and adaptation in vertebrate photoreceptors. Vision Res 30: 1923-1948.

15. Detwiler PB, Ramanathan S, Sengupta A, Shraiman BI (2000) Engineering aspects of enzymatic signal transduction: photoreceptors in the retina. Biophys J 79: 2801-2817.

16. Gray-Keller MP, Detwiler PB (1994) The calcium feedback signal in the phototransduction cascade of vertebrate rods. Neuron 13: 849-861.

17. Korenbrot JI, Miller D (1989) Cytoplasmic free calcium concentration in dark-adapted retinal rod outer segments. Vision Res 29: 939-948.

18. Lagnado L, Cervetto L, McNaughton PA (1992) Calcium homeostasis in the outer segments of retinal rods from the tiger salamander. J Physiol 455: 111-142.

19. McCarthy ST, Younger JP, Owen WG (1994) Free calcium concentrations in bullfrog rods determined in the presence of multiple forms of Fura-2. Biophys J 67: 2076-2089.

20. Ratto GM, Payne R, Owen WG, Tsien RY (1988) The concentration of cytosolic free calcium in vertebrate rod outer segments measured with fura-2. J Neurosci 8: 3240-3246.

21. Sampath AP, Matthews HR, Cornwall MC, Fain GL (1998) Bleached pigment produces a maintained decrease in outer segment Ca2+ in salamander rods. J Gen Physiol 111: 53-64.

22. Woodruff ML, Sampath AP, Matthews HR, Krasnoperova NV, Lem J, et al. (2002) Measurement of cytoplasmic calcium concentration in the rods of wild-type and transducin knock-out mice. J Physiol 542: 843-854.

23. Caruso G, Khanal H, Alexiades V, Rieke F, Hamm HE, et al. (2005) Mathematical and computational modeling of spatio-temporal signaling in rod phototransduction. IEE Proc Syst Biol 152: 119-137.

24. Holcman D, Korenbrot JI (2004) Longitudinal diffusion in retinal rod and cone outer segment cytoplasm: the consequence of cell structure. Biophys J 86: 2566-2582.

25. Olson A, Pugh ENJ (1993) Diffusion coefficient of cyclic GMP in salamander rod outer segments estimated with two fluorescent probes. Biophys J 65: 1335-1352.

26. Carter-Dawson LD, Lavail MM (1979) Rods and cones in the mouse retina. J Comp Neur 188: 245-262.

27. Fotiadis D, Liang Y, Filipek S, Saperstein DA, Engel A, et al. (2003) Rhodopsin dimers in native disc membranes. Nature 421: 127-128.

28. Reilander H, Achilles A, Friedel U, Maul G, Lottspeich F, et al. (1992) Primary structure and functional expression of the Na/Ca,K-exchanger from bovine rod photoreceptors. EMBO J 11: 1689-1695.

29. Schnetkamp PP (1990) Cation selectivity of and cation binding to the cGMP-dependent channel in bovine rod outer segment membranes. J Gen Physiol 96: 517-534.

30. Liang Y, Fotiadis D, Maeda T, Maeda A, Modzelewska A, et al. (2004) Rhodopsin signaling and organization in heterozygote rhodopsin knockout mice. J Biol Chem 279: 48189-48196.

31. Lyubarsky AL, Daniele LL, Pugh ENJ (2004) From candelas to photoisomerizations in the mouse eye by rhodopsin bleaching in situ and the light-rearing dependence of the major components of the mouse ERG. Vision Res 44: 3235-3251.

32. Chen CK, Burns ME, Spencer M, Niemi GA, Chen J, et al. (1999) Abnormal photoresponses and light-induced apoptosis in rods lacking rhodopsin kinase. Proc Natl Acad Sci USA 96: 3718-3722.

33. Fan J, Woodruff ML, Cilluffo MC, Crouch RK, Fain GL (2005) Opsin activation of transduction in the rods of dark-reared Rpe65 knockout mice. J Physiol 568: 83-95.

34. Krispel CM, Chen CK, Simon MI, Burns ME (2003) Novel form of adaptation in mouse retinal rods speeds recovery of phototransduction. J Gen Physiol 122: 703-712.

35. Krispel CM, Chen CK, Simon MI, Burns ME (2003) Prolonged photoresponses and defective adaptation in rods of Gbeta5-/- mice. J Neurosci 23: 6965-6971.

36. Mendez A, Burns ME, Roca A, Lem J, Wu LW, et al. (2000) Rapid and reproducible deactivation of rhodopsin requires multiple phosphorylation sites. Neuron 28: 153-164.

37. Mendez A, Burns ME, Sokal I, Dizhoor AM, Baehr W, et al. (2001) Role of guanylate cyclase-activating proteins (GCAPs) in setting the flash sensitivity of rod photoreceptors. Proc Natl Acad Sci USA 98: 9948-9953.

38. Mendez A, Chen J (2002) Mouse models to study gcap functions in intact photoreceptors. Adv Exp Med Biol 514: 361-388.

39. Wang X Z, Wen H, Ablonczy Z, Crouch RK, Makino CL, et al. (2005) Enhanced shutoff of phototransduction in transgenic mice expressing palmitoylation-deficient rhodopsin. J Biol Chem 280: 24293-24300.

40. Woodruff ML, Lem J, Fain GL (2004) Early receptor current of wild-type and transducin knockout mice: photosensitivity and light-induced Ca2+ release. J Physiol 557: 821-828.

41. Xu J, Dodd RL, Makino CL, Simon MI, Baylor DA, et al. (1997) Prolonged photoresponses in transgenic mouse rods lacking arrestin. Nature 389: 505-509.

42. Schnetkamp P (1989) Na-Ca or Na-Ca-K exchange in rod photoreceptors. Prog Biophys Mol Biol 54: 1-29.

43. Schnetkamp PP (1991) Optical measurements of Na-Ca-K exchange currents in intact outer segments isolated from bovine retinal rods. J Gen Physiol 98: 555-573.

44. Schnetkamp PP, Szerencsei RT, Basu DK (1991) Unidirectional Na+, Ca2+, and K+ fluxes through the bovine rod outer segment Na-Ca-K exchanger. J Biol Chem 266: 198-206.

45. Qin N, Baehr W (1994) Expression and mutagenesis of mouse rod photoreceptor cGMP phosphodiesterase. J Biol Chem 269: 3265-3271.

46. Kennedy MJ, Sowa ME, Wensel TG, Hurley JB (2003) Acceleration of key reactions as a strategy to elucidate the rate-limiting chemistry underlying phototransduction inactivation. Invest Ophthalmol Vis Sci 44: 1016-1022.

47. Krispel CM, Chen D, Melling N, Chen YJ, Martemyanov KA, et al. (2006) RGS expression rate-limits recovery of rod photoresponses. Neuron 51: 409-416.

48. Ramanathan S, Detwiler PB, Sengupta AM, Shraiman BI (2005) G-protein-coupled enzyme cascades have intrinsic properties that improve signal localization and fidelity. Biophys J 88: 3063-3071.

49. Cohen AI (1960) The ultrastructure of the rods of the mouse retina. Am J Anat 107: 23-48.

50. Hamer RD, Nicholas SC, Tranchina D, Liebman PA, Lamb TD (2003) Multiple steps of phosphorylation of activated rhodopsin can account for the reproducibility of vertebrate rod single-photon responses. J Gen Physiol 122: 419-444.

51. Sitaramayya A, Harkness J, Parkes JH, Gonzalez-Oliva C, Liebman PA (1986) Kinetic studies suggest that light-activated cyclic GMP phosphodiesterase is a complex with G-protein subunits. Biochemistry 25: 651-656.

52. Lem J, Krasnoperova NV, Calvert PD, Kosaras B, Cameron DA, et al. (1999) Morphological, physiological, and biochemical changes in rhodopsin knockout mice. Proc Natl Acad Sci USA 96: 736-741.

53. Liang Y, Fotiadis D, Filipek S, Saperstein DA, Palczewski K, et al. (2003) Organization of the G protein-coupled receptors rhodopsin and opsin in native membranes. J Biol Chem 278: 21655-21662.
